# Supplementary material for: Serum amyloid A promotes glycolysis of neutrophils during PD-1 blockade resistance in hepatocellular carcinoma
Source: Nat Commun. 2024 Feb 26;15:1754. doi: 10.1038/s41467-024-46118-w (PMC10897330; doi:10.1038/s41467-024-46118-w)
Supplement: Supplementary file 1 — Supplementary Information [file 41467_2024_46118_MOESM1_ESM.pdf]

Supplementary Information

Serum amyloid A promotes glycolysis of neutrophils during PD-1 blockade resistance in hepatocellular carcinoma

Supplementary Tables

Supplementary Table 1

**Supplementary Table 1. Baseline characteristics of the 52 patients treated with anti-PD-1 immunotherapy.**	
**Category of PD-1**	
pembrolizumab	14
nivolumab	8
sintilimab	8
toripalimab	22
**SAA** (mg/L)	
median (interquartile range)	12.3 (6.7–75.8)
> 20/≤ 20	31/21
**Age** (years)	
median (interquartile range)	52.5 (41.3–59.0)
> 50/≤ 50	28/24
**Sex** (male/female)	48/4
**Hepatitis B virus infection** (absent/present)	7/45
**Cirrhosis** (absent/present)	9/43
**Child-Pugh class** (A/B)	19/33
**ALBI** (1/2)	29/23
**Size** (cm)	
median (interquartile range)	7.0 (4.2-8.3)
> 7/≤ 7	23/29
**Tumor number** (single/multiple)	8/44
**Portal vein invasion** (absent/present)	18/34
**Extrahepatic spread** (absent/present)	31/21

**α-fetoprotein** (ng/mL)	
median (interquartile range)	177.9 (17.8–6247.0)
> 400/≤ 400	29/23
**PIVKA-II** (mAU/mL)	
median (interquartile range)	779.5 (62.5–6564.8)
> 1000/≤ 1000	27/25

Abbreviations: PD-1, programmed death protein-1; SAA, serum amyloid A; ALBI, albumin-bilirubin; PIVKA-II, vitamin K absence or antagonist-II.

Supplementary Table 2

**Supplementary Table 2. Univariate and multivariate analysis of risk factors in the 52 patients treated with anti-PD-1 immunotherapy.**				
	**Univariate**		**Multivariate**	
**Response (PD)**	**OR (95% CI)**	***P*-value**	**OR (95% CI)**	***P*-value**
SAA (threshold 20 mg/L)	19.333 (3.627–103.048)	0.001	19.333 (3.627–103.048)	0.001
Age (threshold 50 years)	0.833 (0.242–2.866)	0.772		
Sex (male vs. female)	0.897 (0.086–9.420)	0.928		
Hepatitis B virus (absent vs. present)	1.100 (0.188–6.445)	0.916		
Child-Pugh class (A vs. B)	0.613 (0.162–2.318)	0.471		
ALBI (1 vs. 2)	0.598 (0.193–1.853)	0.373		
Portal vein invasion (absent vs. present)	0.615 (0.175–2.170)	0.450		
Extrahepatic spread (absent vs. present)	1.150 (0.332–3.983)	0.825		
α-fetoprotein (threshold 400 ng/mL)	0.727 (0.213–2.488)	0.612		
PIVKA-II (threshold 1000 mAU/mL)	0.404 (0.114–1.437)	0.162		
**Overall survival**	**HR (95% CI)**	***P*-value**	**HR (95% CI)**	***P*-value**

SAA (threshold 20 mg/L)	4.564 (1.722–12.096)	0.002	4.997 (1.847–13.517)	0.002
Age (threshold 50 years)	0.694 (0.282–1.709)	0.427		
Sex (male vs. female)	1.940 (0.258–14.566)	0.519		
Hepatitis B virus (absent vs. present)	1.576 (0.364–6.826)	0.543		
Child-Pugh class (A vs. B)	1.811 (0.652–5.029)	0.255		
ALBI (1 vs. 2)	0.673 (0.278–1.629)	0.380		
Portal vein invasion (absent vs. present)	1.708 (0.685–4.257)	0.251		
Extrahepatic spread (absent vs. present)	1.545 (0.585–4.078)	0.380		
α-fetoprotein (threshold 400 ng/mL)	2.853 (1.116–7.295)	0.029	3.196 (1.220–8.373)	0.018
PIVKA-II (threshold 1000 mAU/mL)	2.231 (0.877–5.678)	0.092		
**Progression-free survival**	**HR (95% CI)**	***P*-value**	**HR (95% CI)**	***P*-value**
SAA (threshold 20 mg/L)	3.163 (1.586–6.308)	0.001	3.279 (1.606–6.691)	0.001
Age (threshold 50 years)	0.860 (0.439–1.686)	0.661		
Sex (male vs. female)	1.569 (0.374–6.585)	0.538		
Hepatitis B virus (absent vs. present)	0.946 (0.364–2.643)	0.910		
Child-Pugh class (A vs. B)	1.552 (0.754–3.195)	0.233		
ALBI (1 vs. 2)	0.931 (0.507–1.711)	0.818		

Portal vein invasion (absent vs. present)	1.361 (0.671–2.761)	0.392		
Extrahepatic spread (absent vs. present)	1.414 (0.698–2.866)	0.337		
α-fetoprotein (threshold 400 ng/mL)	2.019 (1.023–3.985)	0.043	2.137 (1.050–4.347)	0.036
PIVKA-II (threshold 1000 mAU/mL)	2.621 (1.307–5.258)	0.007	2.013 (0.994–4.079)	0.052
Cox proportional hazards model test: two-sided with adjustments made for multiple comparisons.				

Supplementary Table 3

**Supplementary Table 3. Baseline characteristics of the 6 patients with snap-freezed fresh core-needle biopsy for RNA-seq.**	
**SAA** (mg/L)	
median (interquartile range)	15.8(9.4–52.1)
> 20/≤ 20	3/3
**Age** (years)	
median (interquartile range)	51.6 (48.6–54.4)
> 50/≤ 50	4/2
**Sex** (male/female)	5/1
**Hepatitis B virus infection** (absent/present)	1/5
**Cirrhosis** (absent/present)	2/4
**Child-Pugh class** (A/B)	5/1
**ALBI** (1/2)	0/6
**Size** (cm)	
median (interquartile range)	5.1 (4.8-5.7)
> 7/≤ 7	2/4
**Tumor number** (single/multiple)	1/5
**Portal vein invasion** (absent/present)	1/5
**Extrahepatic spread** (absent/present)	4/2
**α-fetoprotein** (ng/mL)	
median (interquartile range)	324.6 (123.6–819.3)
> 400/≤ 400	5/1
**PIVKA-II** (mAU/mL)	
median (interquartile range)	125.6 (46.3–562.5)
> 1000/≤ 1000	4/2

Abbreviations: PD-1, programmed death protein-1; SAA, serum amyloid A; ALBI, albumin-bilirubin; PIVKA-II, vitamin K absence or antagonist-II.

Supplementary Table 4

**Supplementary Table 4. Baseline characteristics of the 274 patients treated with anti-PD-1 based combination therapies.**	
**Category of combination**	
TKIs (lenvatinib/sorafenib/regorafenib)	99 (86/8/5)
locoregional therapy (TACE/HAIC)	97 (34/63)
TKIs plus locoregional therapy	78
lenvatinib/sorafenib/regorafenib	71/4/3
TACE/HAIC	28/50
**Category of PD-1**	
pembrolizumab	38
nivolumab	22
sintilimab	82
toripalimab	132
**SAA** (mg/L)	
median (interquartile range)	15.9 (8.3–81.2)
> 20/≤ 20	196/78
**Age** (years)	
median (interquartile range)	54.6 (43.2–62.5)
> 50/≤ 50	147/127
**Sex** (male/female)	241/33
**Hepatitis B virus infection** (absent/present)	44/230
**Cirrhosis** (absent/present)	55/219
**Child-Pugh class** (A/B)	192/82
**ALBI** (1/2)	68/206
**Size** (cm)	
median (interquartile range)	7.6 (5.3-8.9)

> 7/≤ 7	121/153
**Tumor number** (single/multiple)	119/155
**Portal vein invasion** (absent/present)	88/186
**Extrahepatic spread** (absent/present)	179/95
**α-fetoprotein** (ng/mL)	
median (interquartile range)	201.6 (13.5–6893.8)
> 400/≤ 400	183/91
**PIVKA-II** (mAU/mL)	
median (interquartile range)	825.9 (55.9–7021.6)
> 1000/≤ 1000	202/72

Abbreviations: PD-1, programmed death protein-1; TKIs, tyrosine kinase inhibitors; TACE, transarterial chemoembolization; HAIC, hepatic arterial infusion chemotherapy; SAA, serum amyloid A; ALBI, albumin-bilirubin; PIVKA-II, vitamin K absence or antagonist-II.

Supplementary Table 5

**Supplementary Table 5. Baseline characteristics of the 138 patients treated with anti-PD-1 based treatment for validation.**	
**Category of combination**	
TKIs (lenvatinib/sorafenib/regorafenib)	72 (52/19/1)
locoregional therapy (TACE/HAIC)	38 (9/29)
TKIs plus locoregional therapy	28
lenvatinib/sorafenib/regorafenib	22/6/0
TACE/HAIC	7/21
**Category of PD-1**	
pembrolizumab	21
nivolumab	18
sintilimab	38
toripalimab	61
**ALBI** (1/2)	52/86
**Size** (cm)	
median (interquartile range)	7.3 (4.9-9.1)
> 7/≤ 7	75/63
**Tumor number** (single/multiple)	32/106
**Portal vein invasion** (absent/present)	40/98
**Extrahepatic spread** (absent/present)	77/61
**α-fetoprotein** (ng/mL)	
median (interquartile range)	196.3 (15.2–6589.6)
> 400/≤ 400	78/60
**PIVKA-II** (mAU/mL)	
median (interquartile range)	659.3 (72.3–5698.4)
> 1000/≤ 1000	89/49

Abbreviations: PD-1, programmed death protein-1; TKIs, tyrosine kinase inhibitors; TACE, transarterial chemoembolization; HAIC, hepatic arterial infusion chemotherapy; SAA, serum amyloid A; ALBI, albumin-bilirubin; PIVKA-II, vitamin K absence or antagonist-II.

Supplementary Table 6

**Supplementary Table 6. Baseline characteristics of the 160 patients treated with resection.**	
**SAA** (mg/L)	
median (interquartile range)	10.6(4.3–92.6)
> 20/≤ 20	89/71
**Age** (years)	
median (interquartile range)	52.8 (38.6–60.8)
> 50/≤ 50	98/62
**Sex** (male/female)	146/14
**Hepatitis B virus infection** (absent/present)	45/115
**Cirrhosis** (absent/present)	37/123
**Child-Pugh class** (A/B)	119/41
**ALBI** (1/2)	35/125
**Size** (cm)	
median (interquartile range)	4.3 (2.1-6.5)
> 7/≤ 7	69/91
**Tumor number** (single/multiple)	114/46
**Portal vein invasion** (absent/present)	43/117
**Extrahepatic spread** (absent/present)	160/0
**α-fetoprotein** (ng/mL)	
median (interquartile range)	203.6 (20.6–7543.1)
> 400/≤ 400	99/61
**PIVKA-II** (mAU/mL)	
median (interquartile range)	523.6 (45.6–5986.4)
> 1000/≤ 1000	86/74
Abbreviations: PD-1, programmed death protein-1; SAA, serum amyloid A; ALBI,	

albumin-bilirubin; PIVKA-II, vitamin K absence or antagonist-II.

Supplementary Table 7

**Supplementary Table 7. Baseline characteristics of the 68 patients for fresh blood sample.**	
**SAA** (mg/L)	
median (interquartile range)	10.9 (4.7–55.1)
> 20/≤ 20	31/37
**Age** (years)	
median (interquartile range)	51.8 (40.1–61.2)
> 50/≤ 50	35/33
**Sex** (male/female)	61/7
**Hepatitis B virus infection** (absent/present)	11/57
**Cirrhosis** (absent/present)	16/52
**Child-Pugh class** (A/B)	29/39
**ALBI** (1/2)	27/41
**Size** (cm)	
median (interquartile range)	8.5 (5.3-9.8)
> 7/≤ 7	45/23
**Tumor number** (single/multiple)	48/20
**Portal vein invasion** (absent/present)	30/38
**Extrahepatic spread** (absent/present)	39/29
**α-fetoprotein** (ng/mL)	
median (interquartile range)	116.9 (10.9–5893.2)
> 400/≤ 400	31/37
**PIVKA-II** (mAU/mL)	
median (interquartile range)	456.2 (56.3–4862.3)
> 1000/≤ 1000	35/33
Abbreviations: PD-1, programmed death protein-1; SAA, serum amyloid A; ALBI,	

albumin-bilirubin; PIVKA-II, vitamin K absence or antagonist-II.

Supplementary Table 8

Supplementary Table 8. Antibodies, recombinant proteins, chemicals, cell lines, experimental models, critical commercial assays, software, and instrument.			
**Name**	**Supplier**	**Identifier**	**Additional information**
**Antibodies**			
Anti-mouse PD-1 (clone RMP1-14, rat, monoclonal)	Bio X Cell	Cat# BE0146<br>RRID: AB_10949053	Immunotherapy treatment (1 mg/mL)<br>10 mg/kg twice a week for 2 weeks
Anti-mouse SAA (clone 67604, Goat, Polyclonal)	R&D Systems	Cat# AF2948<br>RRID: AB_2182774	Anti SAA treatment (25 µg/mL)<br>5 µg/mouse twice a week for 2 weeks
Anti-Human/Mouse Myeloperoxidase (Goat, Polyclonal)	R&D Systems	Cat# AF3667<br>RRID: AB_2250866	IF (1:50)
Anti-Human IL-6 (clone 6708, mouse, monoclonal)	R&D Systems	Cat# MAB206<br>RRID: AB_2127617	mIHC (1:200)
Anti-mouse SAA1 + SAA2 (clone	Abcam	Cat# ab199030	IHC (1:1000); IF (1:500)

EPR19235, rabbit, monoclonal)		RRID: unregistered yet	
Anti-human SAA1 + SAA2 (clone EPR19550, rabbit, monoclonal)	Abcam	Cat# ab207445<br>RRID: unregistered yet	mIHC (1:1000); IF (1:500); WB (1:5000)
Anti-PD-L1 (clone SP142, rabbit, monoclonal)	Abcam	Cat# ab228462<br>RRID: AB_2827816	mIHC (1:200); IF (1:50)
Anti-PD-L1 (clone D5V3B, rabbit, monoclonal)	Cell Signaling Technology	Cat# 64988S<br>RRID: AB_2799672	IHC (1:200); IF (1:50)
Anti-Stat3 (clone 124H6, mouse, monoclonal)	Cell Signaling Technology	Cat# 9139T<br>RRID: unregistered yet	WB (1:1000)
Anti-Phospho-Stat3 (Tyr705) (clone D3A7 Rabbit, monoclonal)	Cell Signaling Technology	Cat# 9145T<br>RRID: unregistered yet	WB (1:2000)
Anti-β-Actin (clone 13E5, Rabbit, monoclonal)	Cell Signaling Technology	Cat# 4970S<br>RRID: AB_2223172	WB (1:1000)
Anti-PKM2 (clone D78A4, rabbit, monoclonal)	Cell Signaling Technology	Cat# 4053S<br>RRID: AB_1904096	WB (1:1000)
Anti-LDHA (C4B5, rabbit, monoclonal)	Cell Signaling Technology	Cat# 3582S	WB (1:1000)

		RRID: AB_2066887	
Anti-MCT4 (rabbit, polyclonal)	Proteintech	Cat# 22787-1-AP<br>RRID: AB_11182479	WB (1:2000)
Anti-GAPDH (D16H11, rabbit, monoclonal)	Cell Signaling Technology	Cat# 5174S<br>RRID: AB_10622025	WB (1:1000)
Anti-human CD15 (clone MMA + BY87rabbit, monoclonal)	ZSGB-Bio	Cat# ZM-0037<br>RRID: unregistered yet	mIHC (1:200)
Anti-human CD68 (clone KP1, rabbit, monoclonal)	ZSGB-Bio	Cat# ZM-0060<br>RRID: AB_2904190	mIHC (1:200)
Anti-human CD8 (clone SP16, rabbit, monoclonal)	ZSGB-Bio	Cat# ZA-0508<br>RRID: AB_2890107	mIHC (1:200)
Anti-human OSM (clone G-1, mouse, monoclonal)	Santa Cruz Biotechnology	Cat# Sc-390253	WB (1:1000)
Anti-human Arginase 1 (rabbit, polyclonal)	GeneTex	Cat# GTX109242<br>RRID: AB_2036264	WB (1:500)
Anti-IL-1β (clone 3A6, mouse,	Cell Signaling Technology	Cat# 12242S	WB (1:1000)

monoclonal)		RRID: AB_2715503	
Anti-mouse IFN-γ (clone H22, Armenian Hamster, monoclonal)	BioLegend	Cat# 513202<br>RRID: AB_1089144	IF (1:50)
Donkey anti-Goat IgG (H+L) Cross-Adsorbed Secondary Antibody, Alexa Fluor 488	Invitrogen	Cat# A-11055<br>RRID: AB_2534102	IF (1:500)
Donkey anti-Rabbit IgG (H+L) Highly Cross-Adsorbed Secondary Antibody, Alexa Fluor 594	Invitrogen	Cat# A-21207<br>RRID: AB_141637	IF (1:500)
Goat Anti-Mouse IgG H&L (Alexa Fluor 647)	Abcam	Cat# ab150115<br>RRID: AB_2687948	IF (1:500)
Ultra-LEAF Purified anti-human CD28	BioLegend	Cat# 302934<br>RRID: AB_11148949	T cell stimulation (2 ug/mL)
Ultra-LEAF Purified anti-human CD3	BioLegend	Cat# 317326<br>RRID: AB_11150592	T cell stimulation (2 ug/mL)
APC anti-human CD16	BioLegend	Cat# 302012	Flow cytometry (100×)

		RRID: AB_314212	
FITC anti-human CD66b	BioLegend	Cat# 305104<br>RRID: AB_314496	Flow cytometry (100×)
Brilliant Violet 421 anti-human CD274 (B7-H1, PD-L1)	BioLegend	Cat# 329714<br>RRID: AB_2563852	Flow cytometry (100×)
PE anti-human TNF	BioLegend	Cat# 376203<br>RRID: AB_2894502	Flow cytometry (100×)
PE anti-human IFN-γ	BioLegend	Cat# 502508<br>RRID: AB_315233	Flow cytometry (100×)
PE anti-human CD274 (B7-H1, PD-L1)	BioLegend	Cat# 329706<br>RRID: AB_940368	Flow cytometry (100×)
Alexa Fluor 647 Mouse Anti-Human GLUT1	BD Biosciences	Cat# 566580<br>RRID: AB_2869787	Flow cytometry (100×)
Purified anti-mouse CD16/32 Antibody	BioLegend	Cat# 101301<br>RRID: AB_312800	Flow cytometry (100×)
Cell Activation Cocktail (with Brefeldin	BioLegend	Cat#423303	Flow cytometry (100×)

A)			
Alexa Fluor 700 anti-mouse CD45	BioLegend	Cat# 103128<br>RRID: AB_493715	Flow cytometry (100×)
PerCP/Cyanine5.5 anti-mouse CD3	BioLegend	Cat# 100218<br>RRID: AB_1595492	Flow cytometry (100×)
Brilliant Violet 605 anti-mouse CD4	BioLegend	Cat# 100451<br>RRID: AB_2564591	Flow cytometry (100×)
Brilliant Violet 421 anti-mouse CD25	BioLegend	Cat# 102033<br>RRID: AB_10895908	Flow cytometry (100×)
FITC anti-mouse CD3ε	BioLegend	Cat# 100306<br>RRID: AB_312671	Flow cytometry (100×)
Brilliant Violet 510 anti-mouse CD8a	BioLegend	Cat# 100752<br>RRID: AB_2563057	Flow cytometry (100×)
Alexa Fluor 700 anti-mouse/human CD11b	BioLegend	Cat# 101222<br>RRID: AB_493705	Flow cytometry (100×)
APC anti-mouse F4/80	BioLegend	Cat# 123116	Flow cytometry (100×)

		RRID: AB_893481	
Brilliant Violet 650 anti-mouse Ly-6C	BioLegend	Cat# 128049<br>RRID: AB_2800630	Flow cytometry (100×)
PE/Cyanine7 anti-mouse Ly-6G	BioLegend	Cat# 127618<br>RRID: AB_1877261	Flow cytometry (100×)
Brilliant Violet 421 anti-mouse Ly-6G/Ly-6C (Gr-1)	BioLegend	Cat# 108445<br>RRID: AB_2562903	Flow cytometry (100×)
PE anti-mouse CD279 (PD-1)	BioLegend	Cat# 135206<br>RRID: AB_1877231	Flow cytometry (100×)
Brilliant Violet 785 anti-mouse TNF	BioLegend	Cat# 506341<br>RRID: AB_2565951	Flow cytometry (100×)
Alexa Fluor 647 anti-mouse/rat/human FOXP3	BioLegend	Cat# 320013<br>RRID: AB_439749	Flow cytometry (100×)
PE/Cyanine7 anti-mouse CD279 (PD-1)	BioLegend	Cat# 135215<br>RRID: AB_10696422	Flow cytometry (100×)
Brilliant Violet 421 anti-mouse CD69	BioLegend	Cat# 104545	Flow cytometry (100×)

		RRID: AB_2686969	
APC anti-human CD15 (SSEA-1)	BioLegend	Cat# 301908<br>RRID: AB_314200	Flow cytometry (100×)
Zombie NIR Fixable Viability Kit	BioLegend	Cat# 423105	Flow cytometry
Zombie Red Fixable Viability Kit	BioLegend	Cat# 423109	Flow cytometry
**Recombinant proteins**			
Recombinant human SAA	PeproTech	Cat# 300-13	SAA stimulation (1 ug/mL)
Recombinant human IL-6	PeproTech	Cat# 200-06	IL-6 stimulation (2 ug/mL)
Recombinant human IL-8	PeproTech	Cat# 200-08M	IL-8 stimulation (2 ug/mL)
Recombinant Human IL-2	PeproTech	Cat#200-02	IL-2 stimulation (2 ug/mL)
**Chemicals**			
Isoflurane	Piramal	Cat# 56.761.002	Anaesthetic
Napabucasin (BBI608)	Selleck	Cat# S7977	Treatment (1 mg/mL)
Glucose	VWR	Cat# 1.08337.0250	Seahorse
			10 mM glucose
2-deoxyglucose (2-DG)	Sigma-Aldrich	Cat# D8375-5G	Seahorse

			50 mM 2-DG
FX-11	MedChemExpress	Cat# HY-16214	Treatment with 50 mM
TEPP-46	MedChemExpress	Cat#HY-18657	Treatment with 100 μM
OSM-SMI-10B	MedChemExpress	Cat#HY-148692	Treatment with 50 μM
αKG	Sigma-Aldrich	Cat# 13192-04-6	Treatment with 2.5 μg/mL
Phenylmethyl sulfonyl fluoride (PMSF)	Beyotime	Cat# ST506	Protease inhibition (1 mM)
Protease inhibitor cocktail	Cwbio	Cat# CW2200S	Protease inhibition (1 mM)
Phosphatase inhibitor cocktail	Cwbio	Cat# CW2383S	Phosphatase inhibition (1 mM)
Matrigel	Corning	Cat# 356234	Tumor cell implantation
Lenvatinib	Selleck Chemicals	Cat# S1164 & S5240	Treatment (1 mg/mL)
**Cell lines**			
Hepa1-6 (*Mus musculus*)	**Procell**	RRID: CVCL_0327	HCC liver orthotopic mice model
HHL-5 (*Homo sapiens*)	provided by Prof. Bo Li,<br>Sun Yat-sen University		WB
**Experimental models: organism/strains**			
Albumin (Alb)-cre$^{+/+}$ (A)	provided by Xiao-Jun Xia's		*A-cre$^{+/+}$* were provided by Xiao-Jun Xia's

	laboratory, Sun Yat-sen University Cancer Center		laboratory, xxx Cancer Center in a C57BL/6 background
Stat3$^{flox/flox}$ (S)	purchased from Jackson Laboratory		
Saa1$^{-/-}$	Cyagen Biosciences	Cat# S-KO-04183	
Stat3$^{flox/flox}$ & *Alb-cre*$^{+/+}$ and *Stat3*$^{flox/flox}$ & *Alb-cre*$^{-/-}$			A and S were interbred at Sun Yat-sen University Cancer Center
**Commercial assays and kits**			
BCA Protein Assay Kit	Thermo Fisher Scientific	Cat# 71285-3	Protein quantification
Human TH1/TH2 Array 1	RayBiotech	Cat# QAH-TH-1	Quantibody Human Cytokine Arrays
Human serum amyloid A1/SAA1 ELISA Kit	Biodragon	Cat# BDEL-0724-48T	Protein quantification
Human CRP ELISA kit	KeyGEN	Cat# KGC1317-96	Protein quantification
Human Albumin ELISA kit	Neobioscience	Cat# EHC024.48	Protein quantification
Human Oncostatin M ELISA Kit	RayBiotech	Cat# ELH-OSM-1	Oncostatin M test
Human Arginase 1 ELISA kit	RayBiotech	Cat# ELH-ARG1-1	Arginase 1 test

RNA Quick Purification kit	ESscience	Cat# RN001	
HiScript II Q Select RT SuperMix for qPCR (+gDNA wiper)	Vazyme	Cat# R233-01	
ChamQ SYBR qPCR Master Mix	Vazyme	Cat# Q311-02	
MinuteTM Total Protein Extraction Kit	INVENT	Cat# SD-001	
EasySep Human CD8 Positive Selection Kit II	StemCell	Cat# 17853	Human CD8 T cell purification
EasySep Direct Human Neutrophil Isolation Kit	StemCell	Cat# 19666	Human Neutrophil Isolation
Annexin V Apoptosis Detection Kit I	BD Biosciences	Cat# 556547	Apoptosis detection
Seahorse XFe96 FluxPak mini	Agilent	Cat# 102601-100	Seahorse assay
PANO 7-plex IHC kit	Panovue	Cat# 10004100050	mIHC
**Software and Algorithms**			
Analyze 12.0	PerkinElmer		Tumor volume analysis
HALO image analysis	Indica Labs		mIHC analysis
FlowJo	BD Life Sciences	RRID:SCR_008520	Flow cytometry

GraphPad Prism (version 9)	GraphPad Software	RRID:SCR_002798	Statistical analysis
SPSS (version 26.0)	IBM		Statistical analysis
**Instruments**			
In vivo imaging system (IVIS) Spectrum	PerkinElmer		*In vivo* multispectral fluorescence imaging
Vectra Polaris Automated Quantitative Pathology Imaging System	Akoya Biosciences		Human and mice sample multiplexing
LSM980 Confocal laser scanning microscope	ZEISS		Human and mice sample multiplexing
Olympus slide scanner	Olympus	VS120-L100	Slide scanner
XF96 Extracellular Flux analyzer	Seahorse Bioscience	XF96	Seahorse
LSRII SORP	Becton Dickinson		Flow cytometry
Cell Counter	OLYMPUS	Model R1	

Supplementary Table 9

**Supplementary Table 9. Primers for real-time PCR.**		
**Gene name**	**Forward primer**	**Reverse primer**
ALDOA	AGATGAGTCCACTGGGAGCAT	CACGCCCTTGTCTACCTTGAT
ALDOC	GGATGAGTCTGTAGGCAGCAT	GAGTGGTGGTTTCTCCATCAG
ARG1	GCCCAAACCGAAGTCATAG	CAGGAACAGCCACCAATAAG
GAPDH	GGAGTCAACGGATTTGGTCGT	TCTCGCTCCTGGAAGATGGT
GLUT1	CTTTGTGGCCTTCTTTGAAGTG	GACCACACAGTTGCTCCACATAC
LDHA	GATTCCAGTGTGCCTGTATGG	CTACAGAGAGTCCAATAGCCC
IL-1β	TGGTAGCCTCCCTGAAGAAC	AGACTGGGAAACTTTTCCATG
IL-10	CCATAAGGCACAAACTTTCAG	GCAGAAATCAGGAAGGCTG
OSM	GTGAACGGAACAGGTCTC	GAAGGCAGTGACACCATC
PFKFB2	CACCAATACAACCCGGGAGA	GCAGCAATGACATCAGGATCA
PFKFB3	CTCGCATCAACAGCTTTGAGG	TCAGTGTTTCCTGGAGGAGTC
PFKL	CTGTACTCATCAGAGGGCAAG	TGCCAGCATCTTCAGCATGAG
PKM2	TCTGTACCATTGGCCCAGCTT	TGGCTGTGCGCACATTCTTGA

Supplementary Table 10

**Supplementary Table 10. Abbreviations.**	
2DG	2-Deoxy-D-glucose
aHCC	advanced hepatocellular carcinoma
αPD-1	PD-1 antibody
αSAA	SAA antibody
AFP	alpha-fetoprotein
ALDO	aldolase
ARG1	arginase I
AUC	area under the ROC curve
CI	confidence interval
CR	complete response
CRP	C-reactive protein
DAPI	4′,6-diamidino-2-phenylindole
DEGs	differentially expressed genes
DMSO	dimethyl sulfoxide
ECAR	extracellular acidification rate
FACS	fluorescence-activated cell sorting
FPKM	fragments per kilobase of transcript per million mapped reads
GAPDH	glyceraldehyde-3-phosphate dehydrogenase
GLUT1	glucose trans porter-1
HCC	hepatocellular carcinoma
HR	hazard ratio
IFN-γ	interferon-γ
IG	immunoglobulin
IVIS	*in-vivo* imaging system
LDH	lactate dehydrogenase

LDHA	lactate dehydrogenase A
LR	locoregional treatment
mIHC	multiplexed immunohistochemistry
MPO	myeloperoxidase
NEU	neutrophils
NLR	neutrophil to lymphocyte ratio
NPD	non-progressive disease
OR	odds ratio
OS	overall survival
OSM	oncostatin M
PD	progressive disease
PD-1	programmed death protein-1
PD-L1	programmed death protein-1 ligand
PFK	phosphofructokinase
PFS	progression-free survival
PIVKA-II	vitamin K absence or antagonist-II
PK	pyruvate kinase
PKM2	pyruvate kinase M2
PLR	platelet-to-lymphocyte ratio
PR	partial response
p-STAT3	phosphorylated STAT3
qPCR	quantitative real-time PCR
RECIST	Response Evaluation Criteria in Solid Tumors
ROC	receiver operating characteristic
SAA	serum amyloid A
SD	stable disease
ssGSEA	single-sample sequence set enrichment analysis

STAT3	signal transducer and activator of transcription 3
TEPP	tetraethyl Pyrophosphate, a molecule promoting PKM2 tetramer assembly
TKIs	tyrosine kinase inhibitors
TNF	tumor necrosis factor
TMA	tissue microarray
TME	tumor microenvironment
TPM	transcripts per million

Supplementary Figures

Supplementary Fig. 1

a

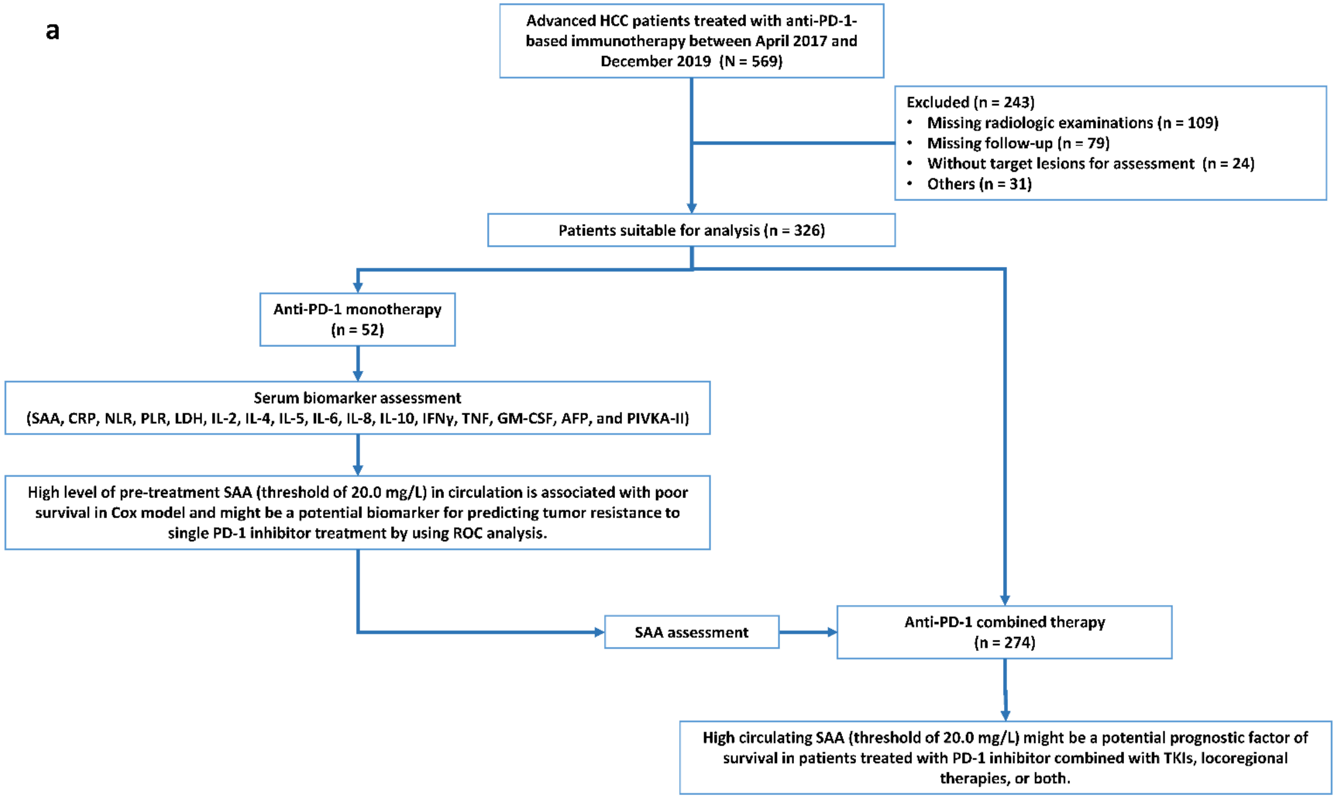

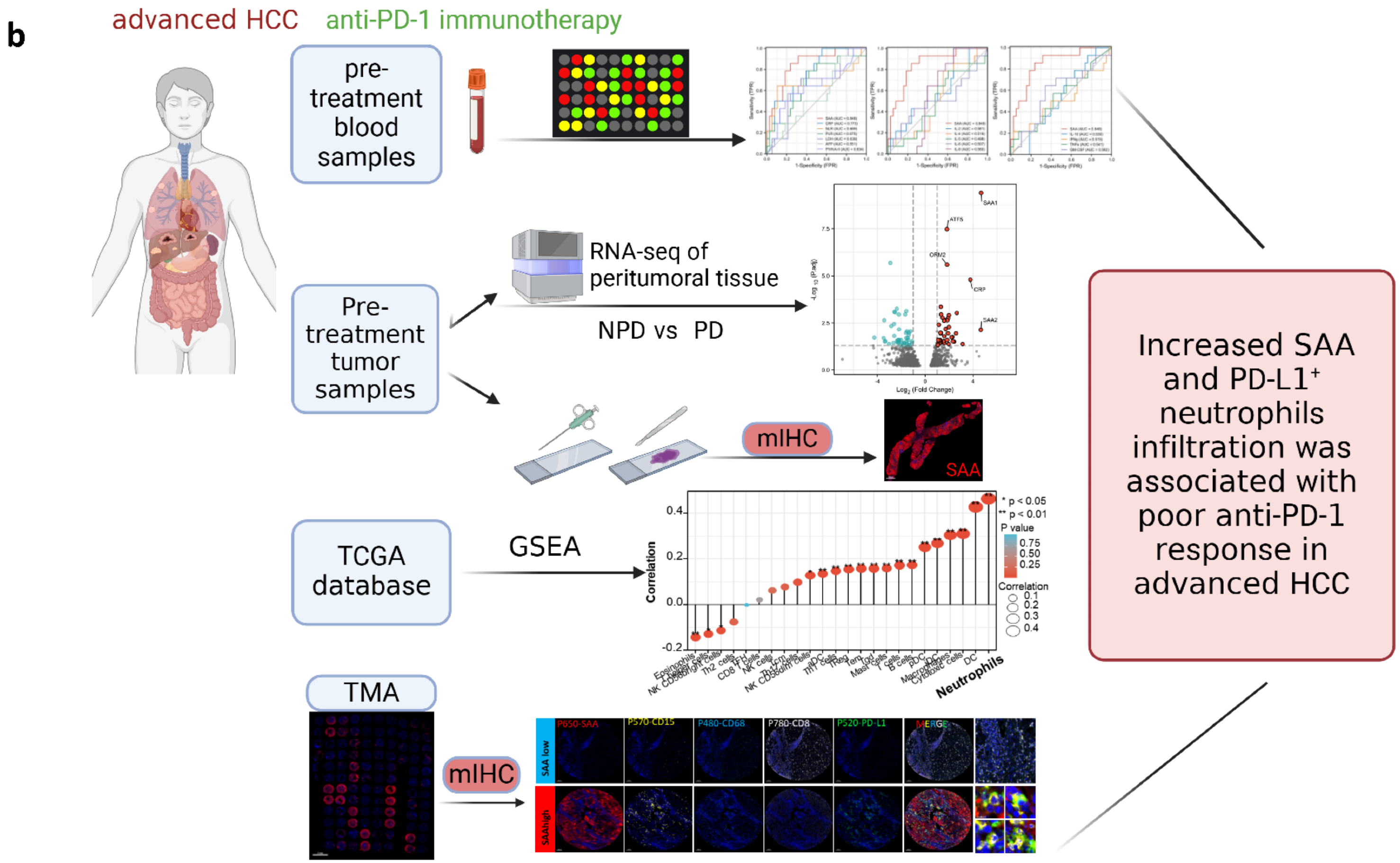
b
advanced HCC
anti-PD-1 immunotherapy
pre-treatment blood samples
Pre-treatment tumor samples
RNA-seq of peritumoral tissue
NPD vs PD
mIHC
SAA
TCGA database
GSEA
Neutrophils
TMA
mIHC
Increased SAA and PD-L1+ neutrophils infiltration was associated with poor anti-PD-1 response in advanced HCC

Supplementary Fig. 1. Study population screening and preliminary assessment of the prognostic value of SAA in patients treated with anti-PD-1 immunotherapy. (a) Flowchart of the study population screening. (b) Study schematic of the correlation between SAA and tumor response to anti-PD-1 immunotherapy in aHCC. First, high circulating SAA before PD-1 blockade is positively associated with the progression of disease by using RECIST (v1.1) radiological assessment criteria. Second, SAA is overexpressed in peritumoral tissues of patients with PD response by using RNA-seq. Third, by analyzing the relationship between local SAA and immune cell infiltration in HCC from TCGA database, it is indicated that neutrophil infiltration is positively associated with SAA levels in local tissues. Additionally, co-expression of SAA and PD-L1^{+} neutrophils is observed in HCC TMA by the mIHC. Give above, we hypothesized whether local SAA drives PD-L1^{+} neutrophils to induce resistance to anti-PD-1 immunotherapy in HCC. Illustrations created with BioRender.com.

Supplementary Fig. 2

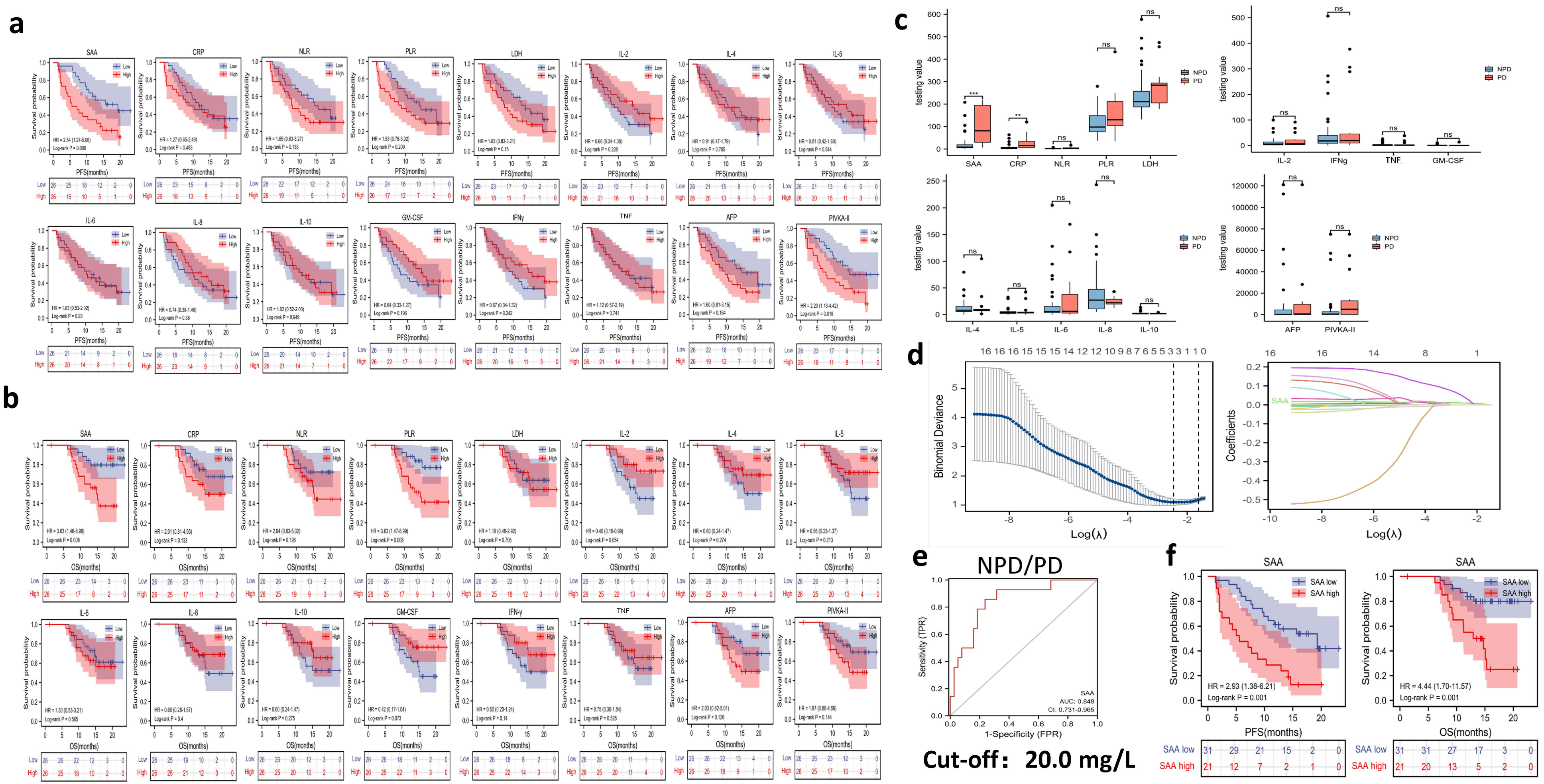

Supplementary Fig. 2. Statistical assessment of the 16 serum biomarkers for aHCC patients treated with single anti-PD-1 immunotherapy. (a-b) Kaplan–Meier curves of survivals show that pre-treatment serum SAA and PIVKA-II are associated with PFS (a); for OS (b), SAA and PLR are potential prognostic factors by log-rank test. n = 52 patients. (c) Besides, there are differences of baseline SAA and CRP levels between patients with NPD and PD tumor response to PD-1 inhibitor. (d) LASSO multivariate analysis further demonstrated that baseline SAA, CRP and NLR were predicting factors to differentiate PD and NPD. Ten time cross-validation for tuning parameter selection in the LASSO model (left), and the LASSO coefficient (right). The linear predictor was defined as (-1.76384186) + SAA × (0.0092548) + CRP × (0.00042891) + NLR × (0.0.07575887). (e) Furthermore, ROC curves are used to identify the best cutoff-value of baseline SAA levels for differentiating patients who are resistant to PD-1 inhibitor. It is suggested that a threshold of 20.0 mg/L of serum SAA prior to anti-PD-1 treatment has the highest predicting value for identifying PD patients (AUC: 0.848). (f) Kaplan–Meier curves of PFS and OS between patients stratifying according to the 20.0 mg/L SAA level show that patients with high level of pre-treatment SAA have worse PFS (HR, 2.93; 95% CI, 1.38– 6.21; $P = 0.001$) and OS (HR, 4.44; 95% CI, 1.70–11.57; $P = 0.001$) than those with low level of SAA. The results are expressed as the mean ± SEM. Statistical data presented in this figure show mean ± SEM. ns indicates $P > 0.05$, $^{**}P < 0.01$, and $^{***}P < 0.001$, by two-sided log-rank test (a, b, f), two-sided Student's *t*-test (c), LASSO multivariate analysis (d), or ROC analyses (e). Source data and exact *P* values are provided as a Source Data file. Illustrations created with BioRender.com.

Supplementary Fig. 3

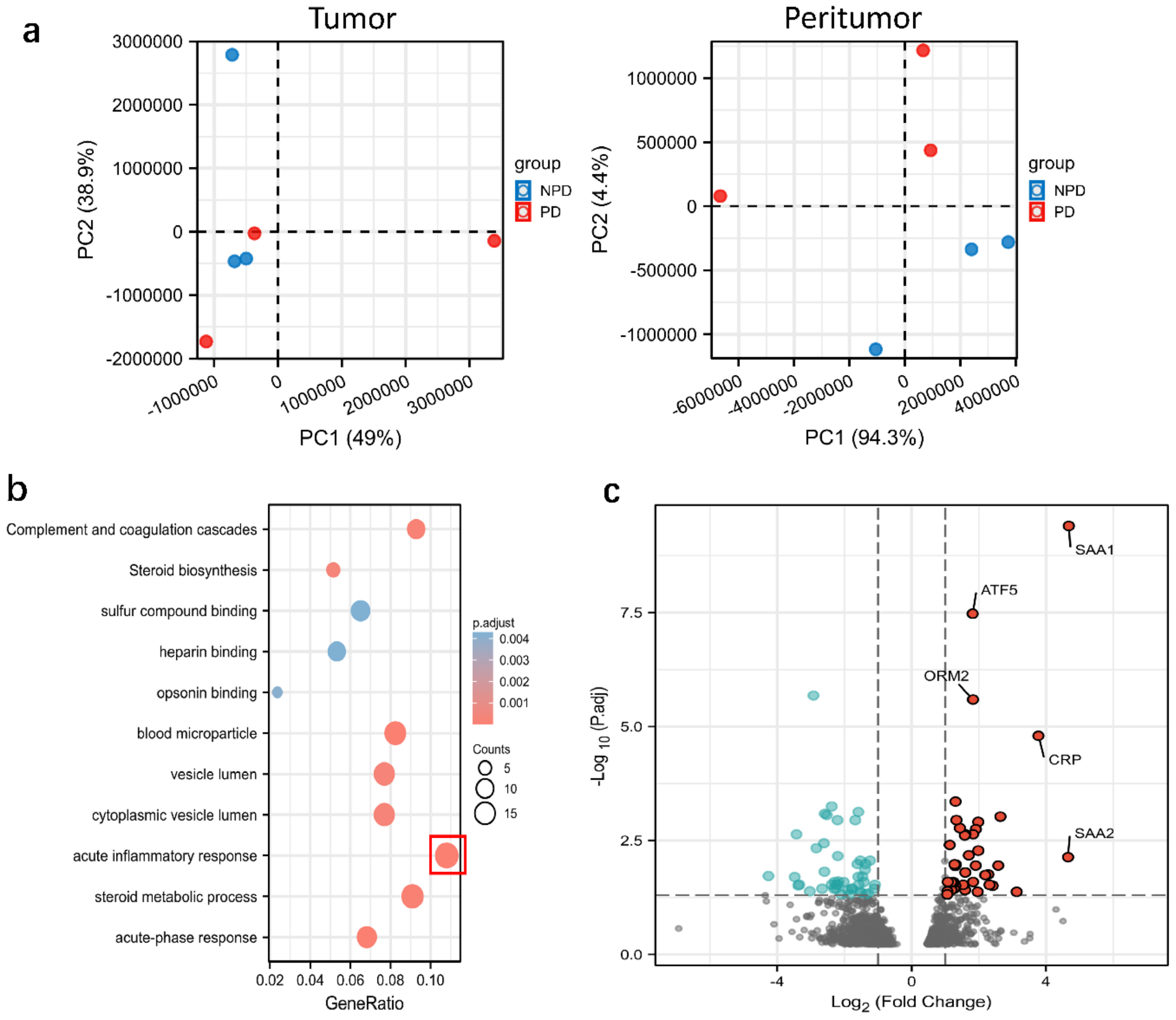

Supplementary Fig. 3. The transcriptome analysis of tumor or peritumor tissue between patient with NPD and PD disease response. (a) RNA-seq used to detect the transcriptome expression in both tumor and peritumor tissues of six patients. n = 3 for each NPD or PD patients. The Principal Component Analysis (PCA) reveals that transcriptome expression of peritumor tissue samples can better differentiate NPD and PD patients treated with PD-1 inhibitor. (b) The upregulation of the acute inflammatory response pathway is uncovered by gene ontology enrichment analysis in peritumor tissues of PD patients. Adjusted *P*-values are annotated by color. The counts of enrichment genes are indicated by circles of different diameters. (c) Volcano plots of the RNA-seq show the different gene expressions in six peritumor biopsies (n = 3 for each NPD or PD case). Acute-phase proteins, including SAA1, SAA2, and CRP, are significantly elevated in PD patients. Red dots represent upregulated genes; blue dots represent downregulated genes; adjusted P <0.05.

Supplementary Fig. 4

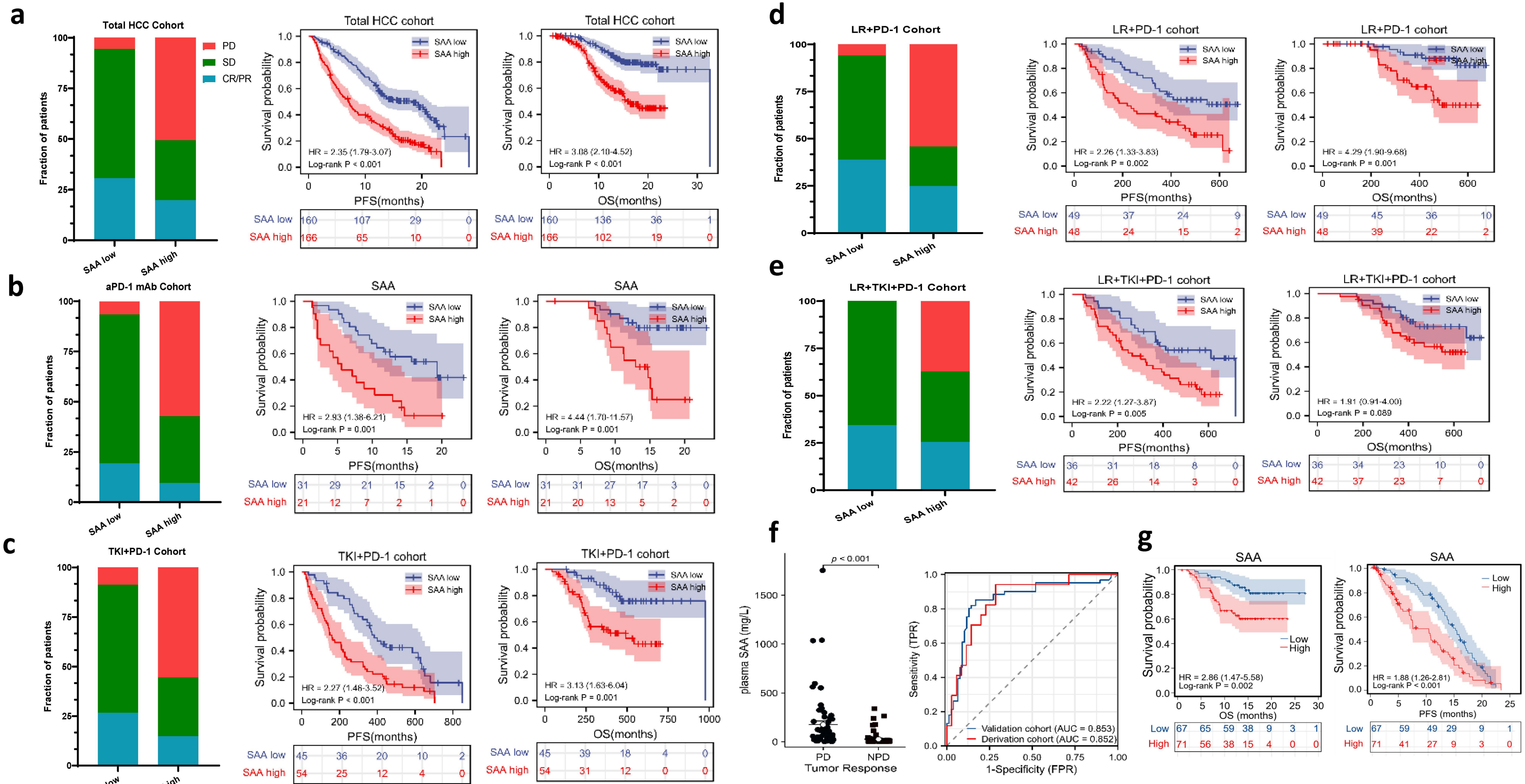

Supplementary Fig. 4. Pre-treatment circulating SAA (cutoff = 20.0 mg/L) is a potential predicting factor of response and prognostic factor of survival. (a-e) In the total 326 aHCC patients treated with PD-1 inhibitor alone or combined with LR, TKIs, or both. (f-g) In the validation cohort of 138 aHCC patients treated with PD-1 inhibitor-based combination treatment. Statistical data presented in this figure show mean ± SEM. Two-sided log-rank test (a, b, c, d, e, g), two-sided Student's *t*-test (left panel of f), or ROC analyses (right panel of f). Source data and exact *P* values are provided as a Source Data file.

Supplementary Fig. 5

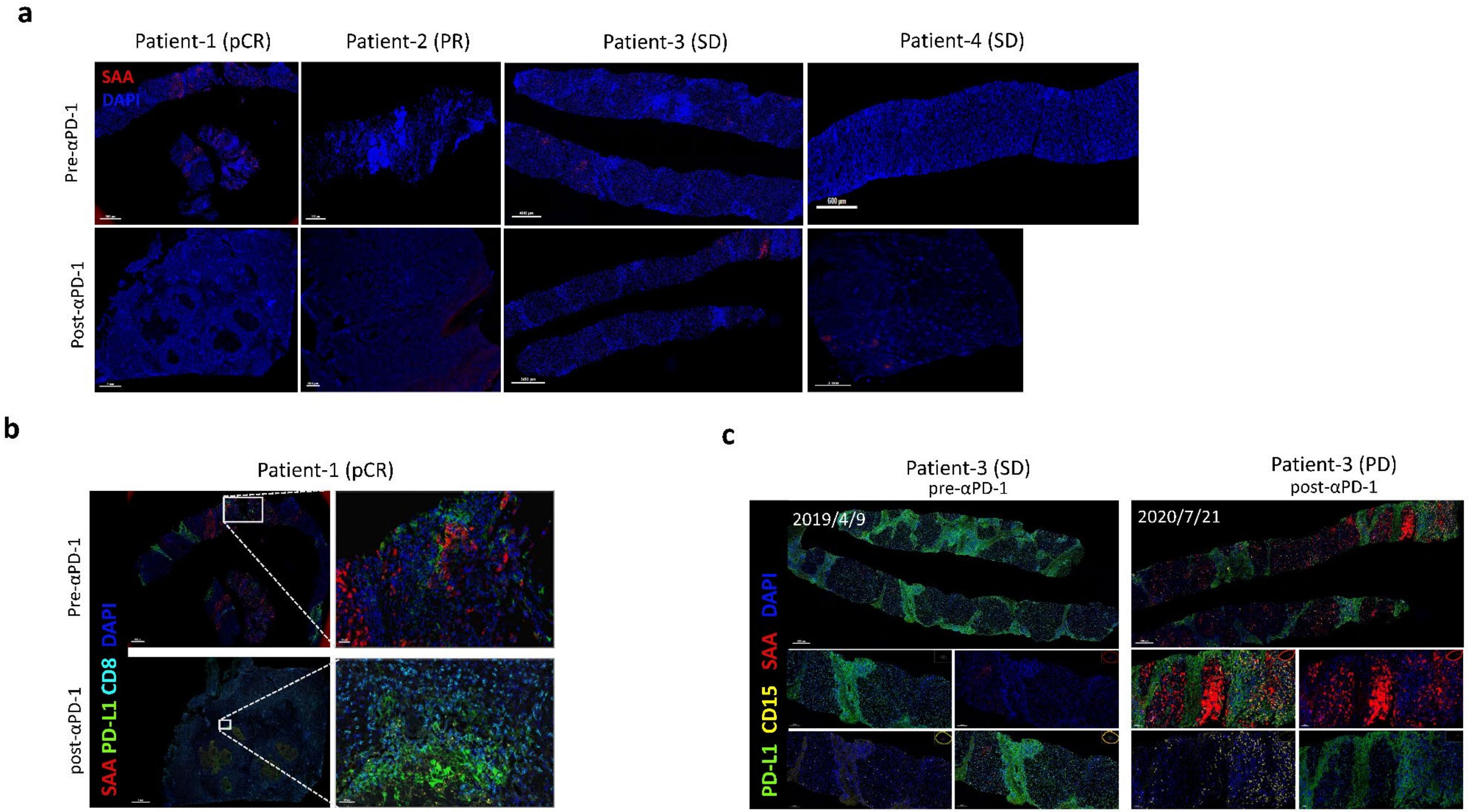

Supplementary Fig. 5. Changes of local SAA expression in aHCC patients with NPD tumor response to anti-PD-1 immunotherapy by mIHC analysis. (a) mIHC images show low expression of local SAA in paired samples of pre- and post-αPD-1 from four patients with NPD tumor response. (b) mIHC images of pCR samples show SAA is reduced after αPD-1 treatment. (c) In a patient with initial SD response, the SAA expression is obviously increased when tumors occur acquired resistance to αPD-1. CD8: brilliant blue, CD15: yellow, DAPI: blue, PD-L1: green, and SAA: red.

Supplementary Fig. 6

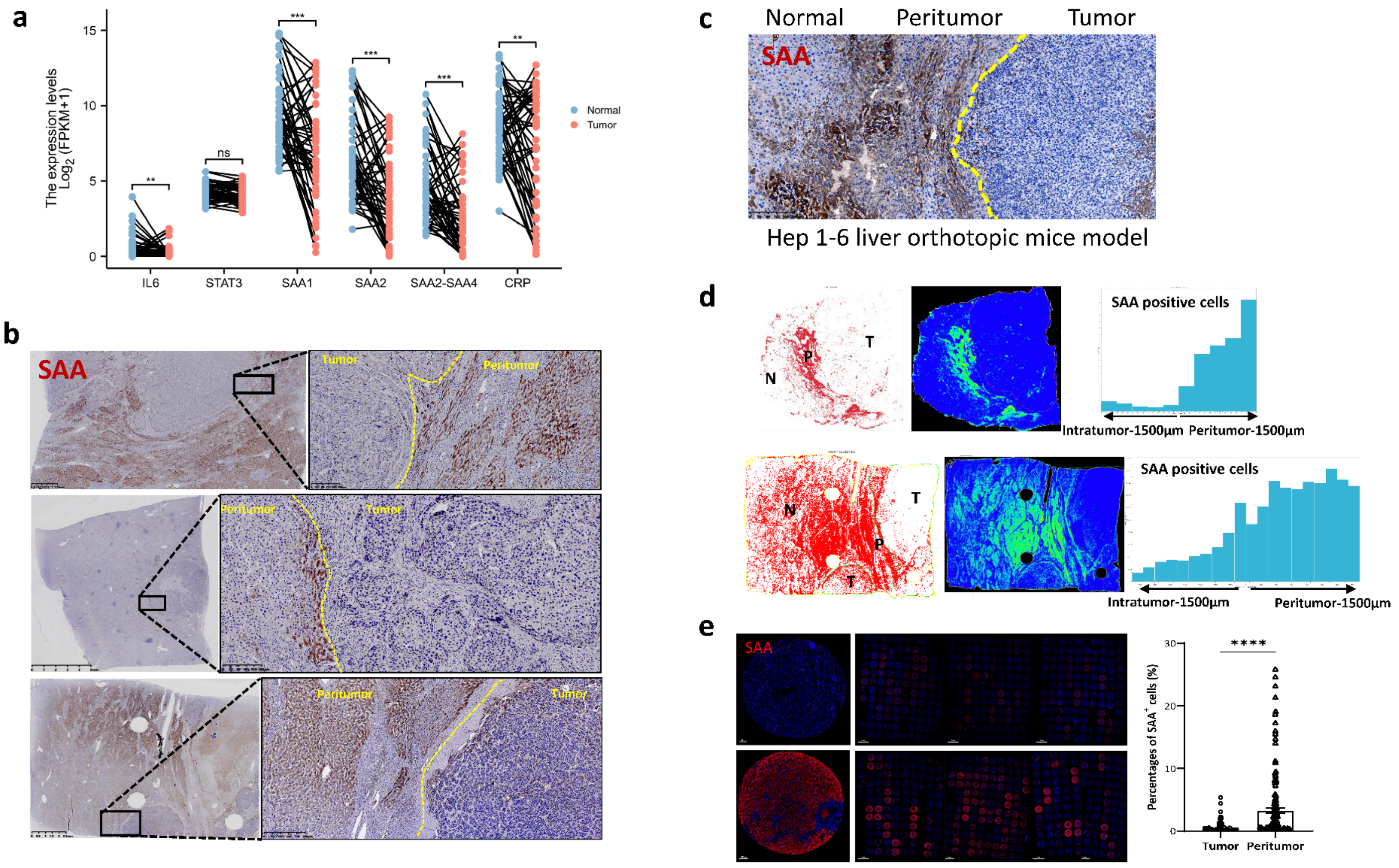

Supplementary Fig. 6. SAA is more expressed in peritumor tissues than in tumor tissues. (a) The mRNA expression of SAA, IL-6, and STAT3 in paired tumor and peritumoral normal tissues of HCC in TCGA database. The results indicate that SAA is considerably more expressed in peritumoral tissues than in tumor tissues, and the mRNA level of IL-6 in the peritumoral tissue of the TCGA database was higher than that in the tumor. (b) Immunohistochemistry protein expression of resected slides also shows that SAA is mainly located in the peritumor area but not in the intertumoral tissue. (c) H&E images show that SAA expression is also mainly located in the peritumor area in HCC mice model. (d) mIHC images show that SAA protein expression is enriched in tumor margin and peritumor tissues. The infiltration analysis by HALO software shows the spatial distribution of SAA in resected HCC specimens. SAA positive cells mainly locate outside the tumor-edge at 1500 µm (count cells every 100–200 µm). (e) In paired tumor and peritumor TMA, the count of SAA positive cells in mIHC images (blue: DAPI; red: SAA) is significantly higher in peritumor than tumor. Statistical data presented in this figure show mean ± SEM. ns indicates $P > 0.05$, $^{**}P < 0.01$, $^{***}P < 0.001$, and $^{****}P < 0.0001$, by two-sided paired *t*-test (a, e). Source data and exact *P* values are provided as a Source Data file.

Supplementary Fig. 7

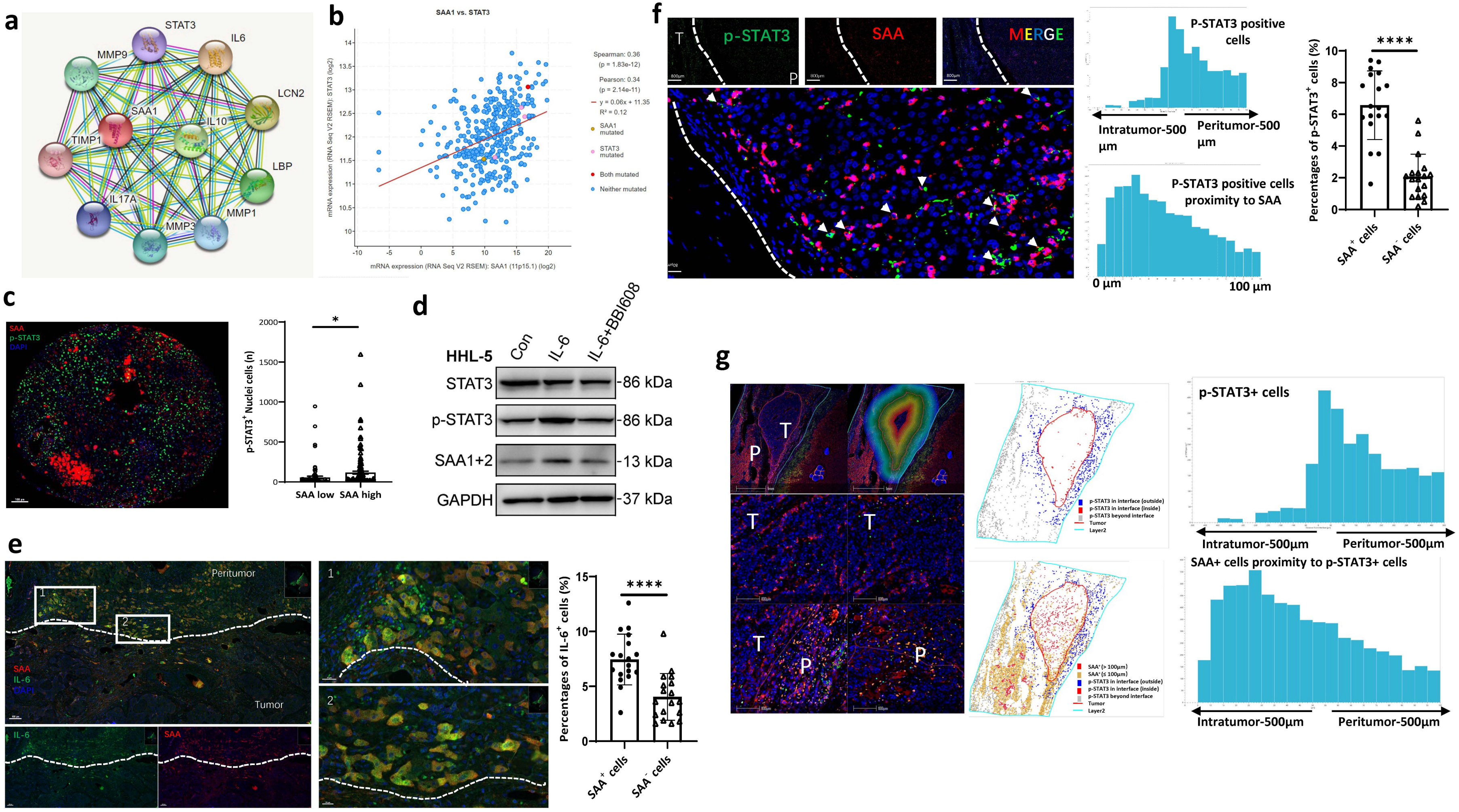

Supplementary Fig. 7. Peritumoral SAA production mainly relies on the activation of STAT3 signaling in hepatocytes. (a) Co-expression protein-protein interaction (PPI) networks show SAA is related to STAT3 by String website analysis. (b) Assessment of mRNA expression shows that there is a positive correlation between SAA and STAT3 in the TCGA database. n = 424 patients. (c) p-STAT3 positive nuclei counts in the mIHC images of the SAA high group are higher than that of the SAA low group in HCC TMA. n = 78, 145 patients separately. (d) Western blot shows that both p-STAT3 and SAA1+2 expression in HHL-5 cells is remarkably upregulated after treatment with IL-6 (20 ng/mL), while the stimulating effect of IL-6 on p-STAT3 and SAA1+2 can be prevented after STAT3 inhibition by napabucasin (BBI608; 0.1 μmol/L). n = 3 independent samples. (e) The mIHC shows the co-expression of SAA and IL-6 in the hepatocytes of the peritumor area from resected HCC specimens. n = 18 patients for each group. (f) The mIHC shows the co-expression of SAA and p-STAT3 in the peritumor tissue from resected HCC specimens (red: SAA positive cells; green: p-STAT3 positive cells; white arrow: dual-positive cells). The spatial cell diagram of HALO analysis shows the relationship of spatial distribution between SAA^+ and p-$STAT3^+$ cells. The infiltration analysis represents the spatial distribution of p-STAT3 positive cells in HCC specimens (right-upper diagram). The distance between p-$STAT3^+$ and SAA^+ cells range from 0 to 50 micrometers (right-lower diagram). n = 18 patients for each group. (g) mIHC images show that SAA^+ and p-$STAT3^+$ cells are enriched in peritumor tissues. The infiltration analysis by HALO software shows that p-$STAT3^+$ cells mainly located outside tumor edge 500 μm (count cells every 100–200 μm). Statistical data presented in this figure show mean ± SEM. *P < 0.05, and ****P < 0.0001, by two-sided Pearson correlation analysis (b), or two-sided Student's *t*-test (c, e, f). Source data and exact *P* values are provided as a Source Data file.

Supplementary Fig. 8

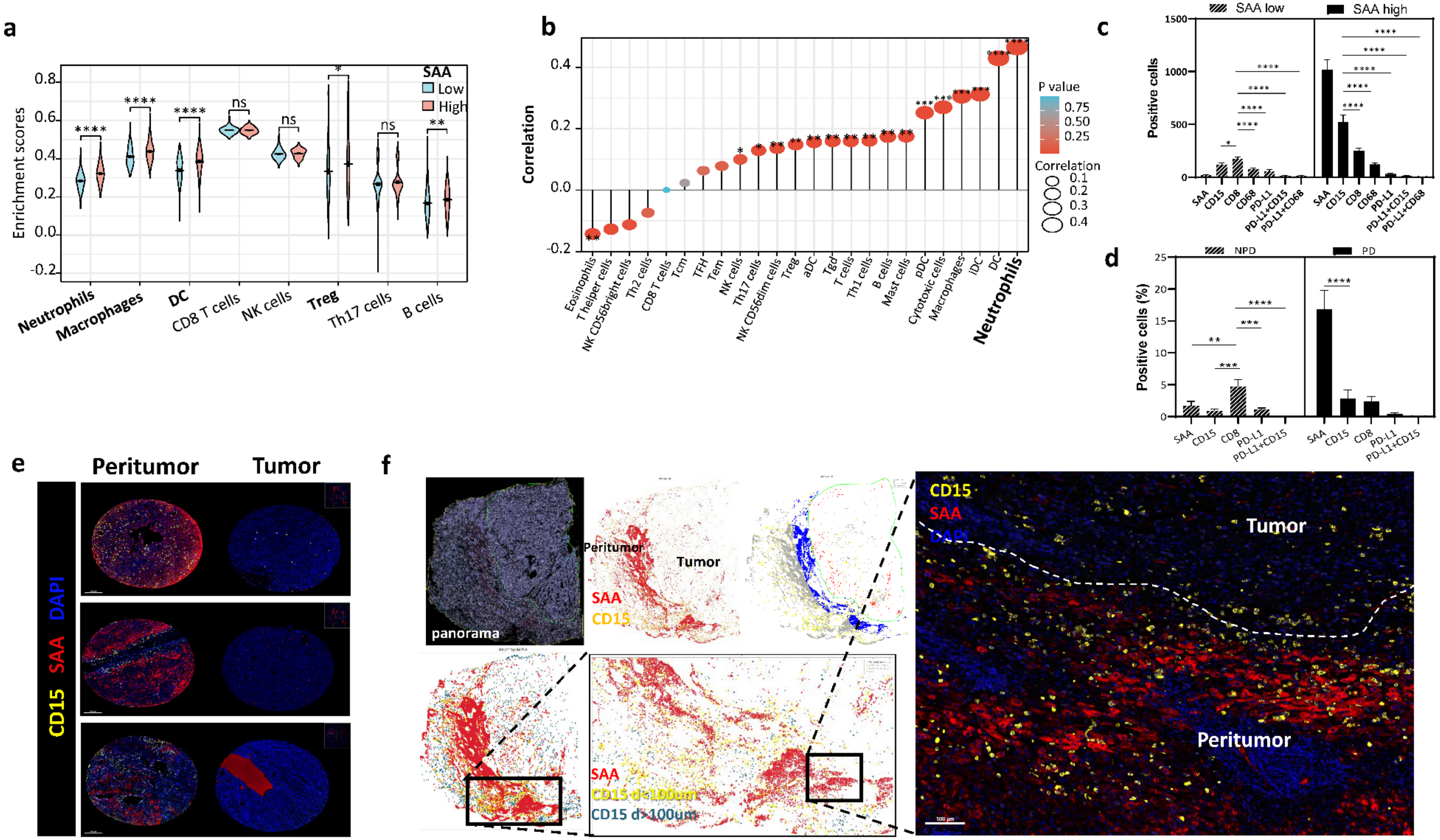

Supplementary Fig. 8. Association between SAA level in tumor tissue and immune cells infiltration in HCC. (a) By ssGSEA analysis, the enrichment levels of multiple immune cells in HCC are compared between high expression and low expression of SAA in tumor tissue from TCGA datasets. Infiltration of neutrophils, macrophages, DC and regulated T cells are elevated in the high SAA expression group compared to the low SAA expression group. (b) A series of correlation coefficients between different immune cells and expression level of SAA in tumor tissue are ranked by using ssGSEA analysis. Neutrophils rank at the top of the positive correlation coefficient list. (c) Count of multiple infiltrating immune cells with specific marker are compared between the SAA-high group (n = 174 patients) and SAA-low group (n = 173 patients). (d) Counts of multiple infiltrating immune cells with the specific marker are compared between the NPD (n = 15 patients) and PD (n = 5 patients) groups. (e) mIHC images of paired peritumor and tumor tissue array show the simultaneous detection of DAPI (blue), SAA (red), and CD15 (yellow). (f) mIHC image shows the distribution of SAA (red) and $CD15^+$ (yellow, expressed on neutrophils) in TME by HALO software. Statistical data presented in this figure show mean ± SEM. ns indicates $P > 0.05$, $^*P < 0.05$, $^{**}P < 0.01$, $^{***}P < 0.001$, and $^{****}P < 0.0001$, by two-sided paired *t*-test (a), two-sided Spearman's rank correlation analysis (b), or one-way ANOVA (c, d). Source data and exact *P* values are provided as a Source Data file.

Supplementary Fig. 9

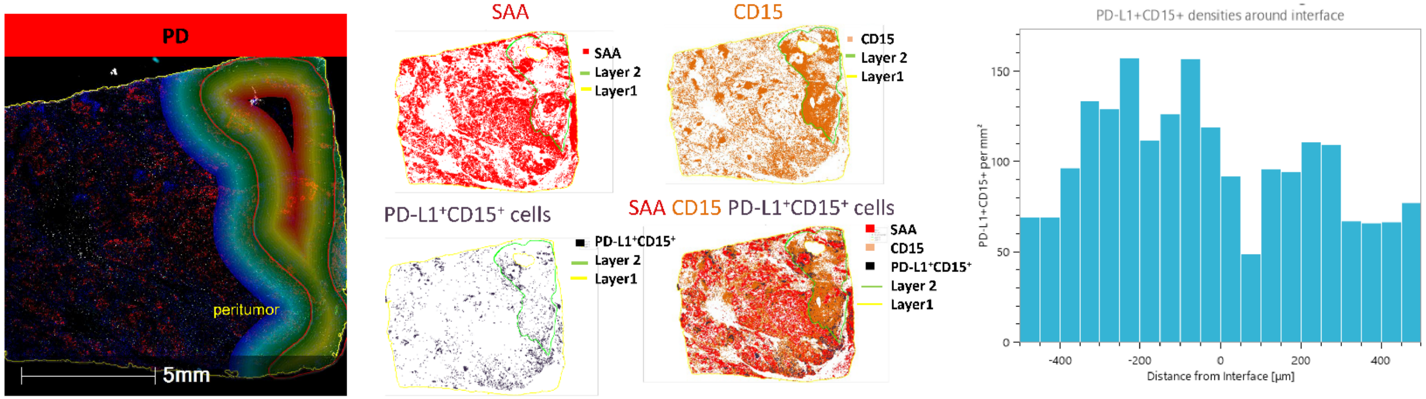

Supplementary Fig. 9. The association between SAA and PD-L1$^+$ neutrophils infiltration in peritumor tissue of PD patients. mIHC image shows the distribution of SAA and PD-L1$^+$CD15$^+$ cells in TME of PD patients. An IHC array also shows the location of SAA, CD15, PD-L1$^+$ neutrophils, and SAA CD15 PD-L1$^+$ NEU, respectively. The infiltration analysis by HALO software shows that the quantity of PD-L1$^+$ neutrophils is enriched in the peritumoral region. CD15: orange, PD-L1$^+$CD15$^+$: black, SAA: red.

Supplementary Fig. 10

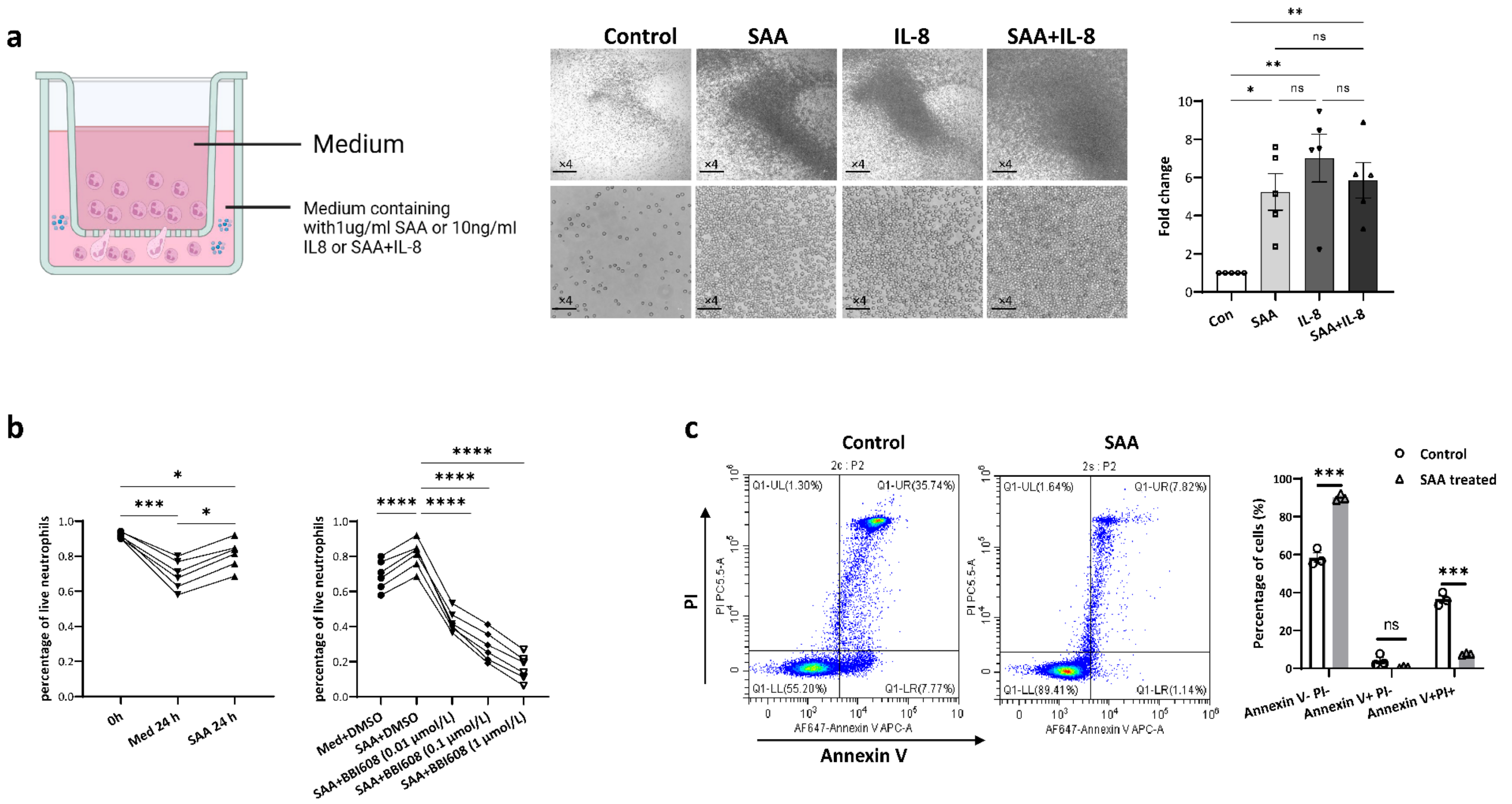

Supplementary Fig. 10. SAA promotes the accumulation and survival of neutrophils in HCC. (a) Schematic diagram (left) and light field (middle) images show that neutrophils pass into the lower chamber in each group. The histogram (right) shows the quantity of neutrophils passed into the lower chamber. SAA (1 μg/mL) had a stronger recruitment effect on neutrophils compared with IL-8 (10 ng/mL). n = 5 biologically independent samples. (b) Neutrophils were unexposed (Med group) or exposed to SAA (1 μg/mL) for 24 h, and the left plot shows that SAA could maintain the viability of neutrophils purified from peripheral blood of HCC patients, compared to that of the Med group. The right plot shows that the addition of BBI608 (napabucasin, a small molecular STAT3 inhibitor) into the SAA group can decrease the effect of SAA and decrease the viability of neutrophils, and this inhibitory role depends on the concentration of BBI608. n = 6 biologically independent samples. (c) Flow cytometry images and histograms show that the apoptosis of neutrophils is inhibited by SAA. n = 3 biologically independent samples. Statistical data presented in this figure show mean ± SEM. ns indicates $P > 0.05$, *$P < 0.05$, **$P < 0.01$, ***$P < 0.001$, and ****$P < 0.0001$, by one-way ANOVA (a, b), or two-sided Student's *t*-test (c). Source data and exact *P* values are provided as a Source Data file. Illustrations created with BioRender.com.

Supplementary Fig. 11

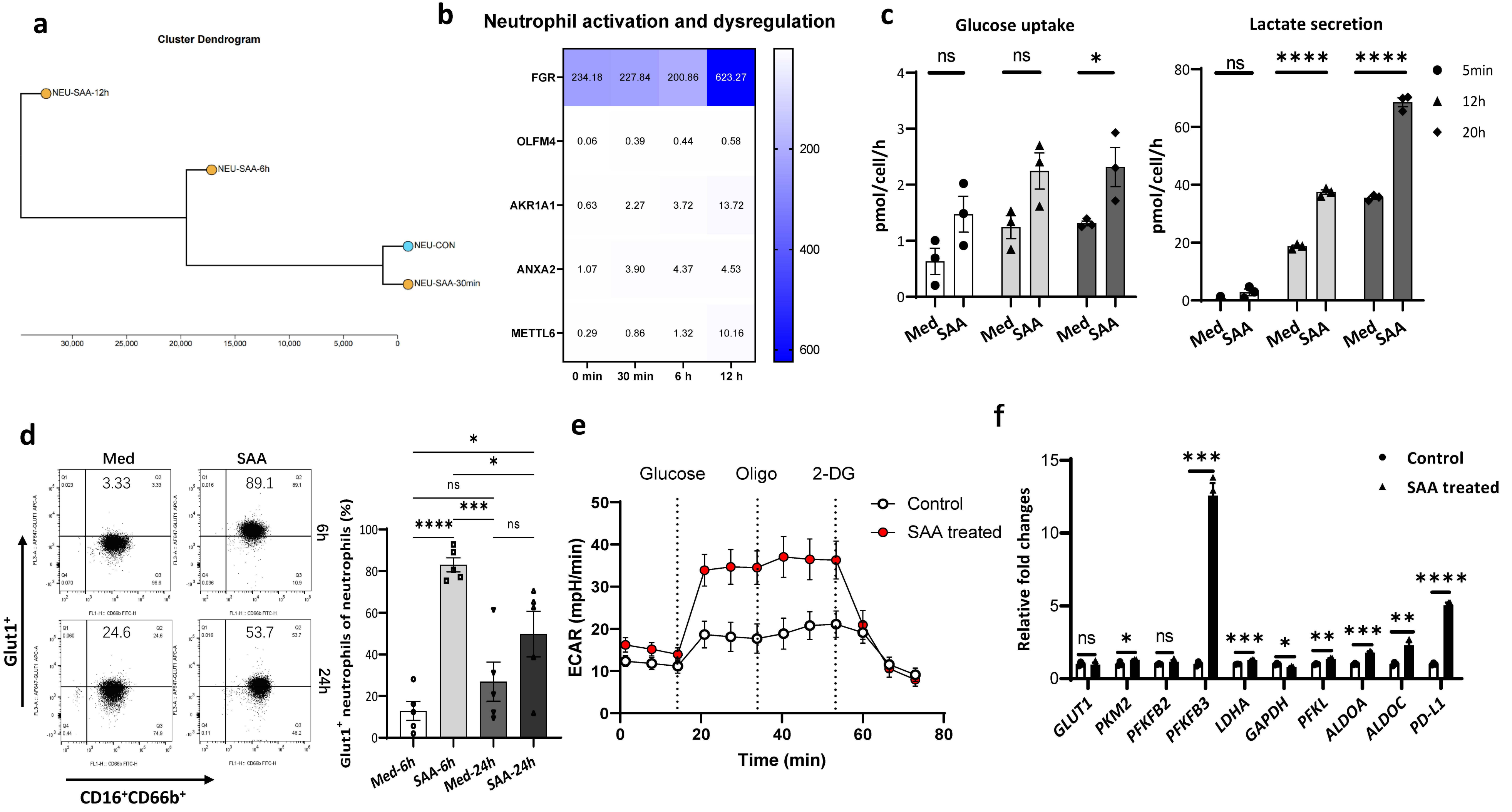

Supplementary Fig. 11. SAA mediates glycolytic activation on neutrophils. (a) Cluster dendrogram of neutrophils exposed to SAA at different timepoints describes the gene clustering level of each group. (b) Heat maps show the expression kinetics of neutrophil activation and dysregulation-related genes, measured by FRKM values from RNA-seq. (c) The histogram shows that neutrophil glucose uptake and lactate secretion are significantly elevated after SAA (1 µg/mL) stimulation for 20 hours by YSI biochemical analyzer. n = 3 biologically independent samples. (d) The levels of Glut1 expression in neutrophils were determined by flow cytometry after being exposed to SAA (1 µg/mL) for 6 and 24 hours. Histogram shows that SAA-induced Glut1 expression on neutrophils occurs at the early stage (6 hours) of stimulation. n = 5 biologically independent samples. (e) Seahorse extracellular flux analysis of the ECAR of neutrophils treated by SAA (1 µg/mL) for 1 hour shows that SAA-treated neutrophils exhibited significantly higher glycolytic capacity. n = 5 biologically independent samples. (f) Neutrophils purified from peripheral blood healthy donors are treated with SAA (1 µg/mL) for 0.5, 6, and 12 hours. The levels of glycolysis-related gene expression were quantified by qPCR. The mRNA expression of *PD-L1* and glycolytic enzymes including *PKM2, PFKFB3, LDHA, GADPH, PFKL, ALDOA, ALDOC,* and *ALDOC* are increased in SAA-treated neutrophils. n = 3 biologically independent samples. Statistical data presented in this figure show mean ± SEM. ns indicates $P > 0.05$, *$P < 0.05$, **$P < 0.01$, ***$P < 0.001$, and ****$P < 0.0001$, by two-sided Student's *t*-test (c, f), or one-way ANOVA (d). Source data and exact *P* values are provided as a Source Data file.

Supplementary Fig. 12

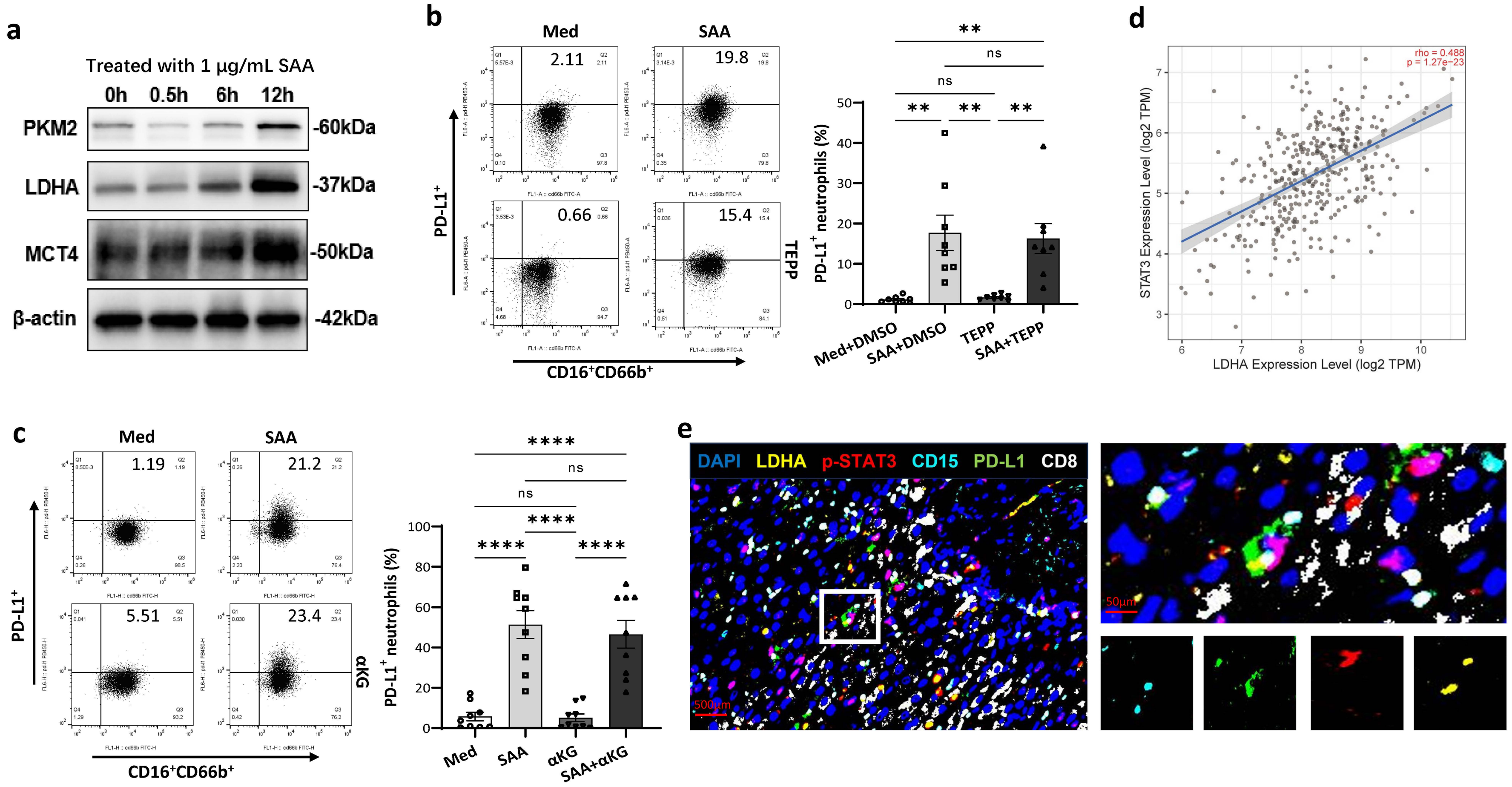

Supplementary Fig. 12. Activation of the LDHA/STAT3 rather HIF-1α/PKM2 axis is involved in the glycolysis-mediated PD-L1 upregulation in SAA-associated neutrophils. (a) The protein expression of PKM2, LDHA, and MCT4 in SAA-treated neutrophils is validated by western blotting analysis. n = 3 independent samples. (b) Flow cytometry images show the proportion of PD-L1$^+$ neutrophils in four groups, among which the neutrophils are exposed to medium (Med+DMSO) or SAA in the absence or presence of TEPP (100 µmol/L) for 12 h. n = 8 biologically independent samples. There is no significant difference of PD-L1$^+$ neutrophils between the SAA+DMSO and SAA+TEPP groups. (c) Flow cytometry images show the proportion of PD-L1$^+$ neutrophils in four groups, among which the neutrophils are exposed to medium (Med+DMSO) or SAA in the absence or presence of aKG (2.5 µg/ml) for 12 h. n = 9 biologically independent samples. There is no significant difference in PD-L1$^+$ neutrophils between the SAA+DMSO and SAA + aKG groups. (d) Expression of LDHA is positively correlated with that of STAT3 in LIHC in the TIMER 2 database. (e) The six-plex mIHC assay shows the co-expression of LDHA (yellow), p-STAT3 (red), and PD-L1 (green) in CD15$^+$ (blue) neutrophils from HCC peritumoral specimens, and located close to CD8$^+$ T cells in the HCC peritumoral area. Statistical data presented in this figure show mean ± SEM. ns indicates $P > 0.05$, $^{**}P < 0.01$, and $^{****}P < 0.0001$, by one-way ANOVA (b, c), or two-sided Pearson correlation analysis (d). Source data and exact *P* values are provided as a Source Data file.

Supplementary Fig. 13

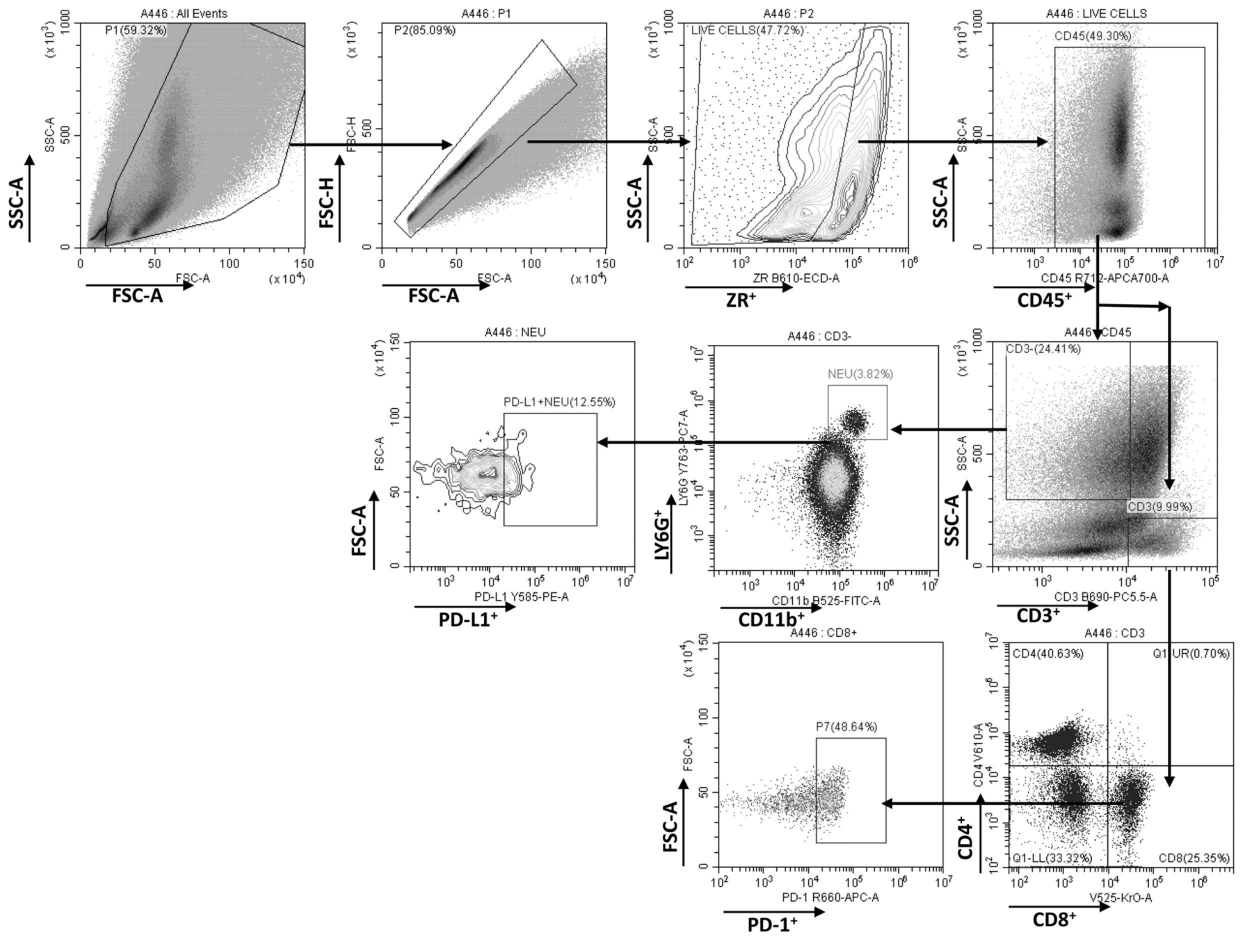

Supplementary Fig. 13. The gate strategy of flow cytometry in HCC mice model.

Supplementary Fig. 14

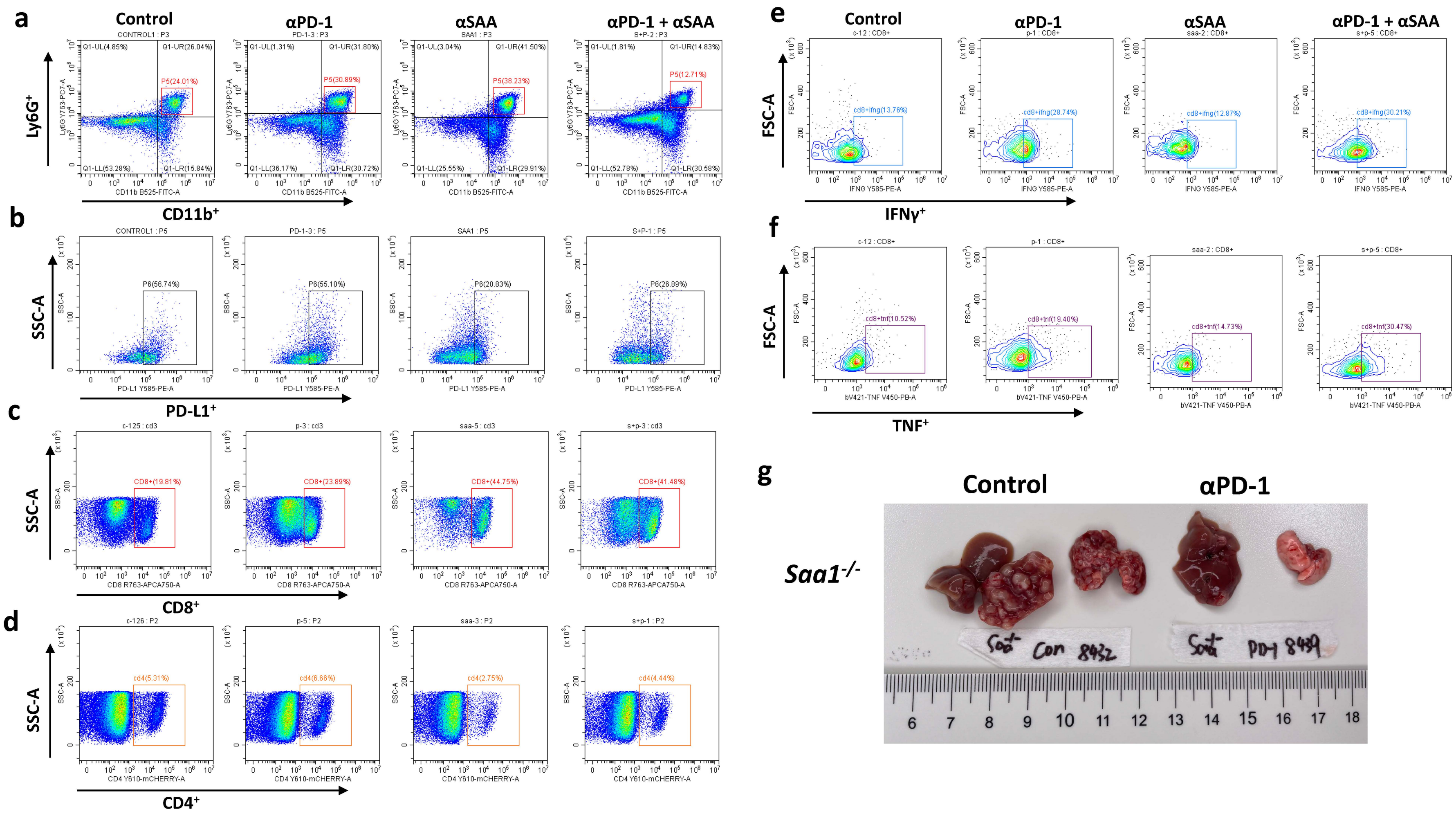

Supplementary Fig. 14. Both SAA protein inhibition and *SAA* gene ablation can enhance the anti-tumor activity of αPD-1 in orthotopic HCC mice models. (a-f) Flow cytometry images show the parent percentage of peritumoral infiltration of $CD11b^+Ly6G^+$ cells (a), $PD\text{-}L1^+CD11b^+Ly6G^+$ cells (b), $CD45^+CD3^+CD8^+$ cells (c), $CD45^+CD3^+CD4^+$ cells (d), $IFNg^+CD8^+$ cells (e), and TNF^+CD8^+ cells (f) in treatment groups including the control, αPD-1, αSAA1+2, or the combination of αPD-1 and αSAA1+2. (g) αPD-1 can actively reduce the tumor burden in the $Saa1^{-/-}$ orthotopic HCC mice model.

Supplementary Fig. 15

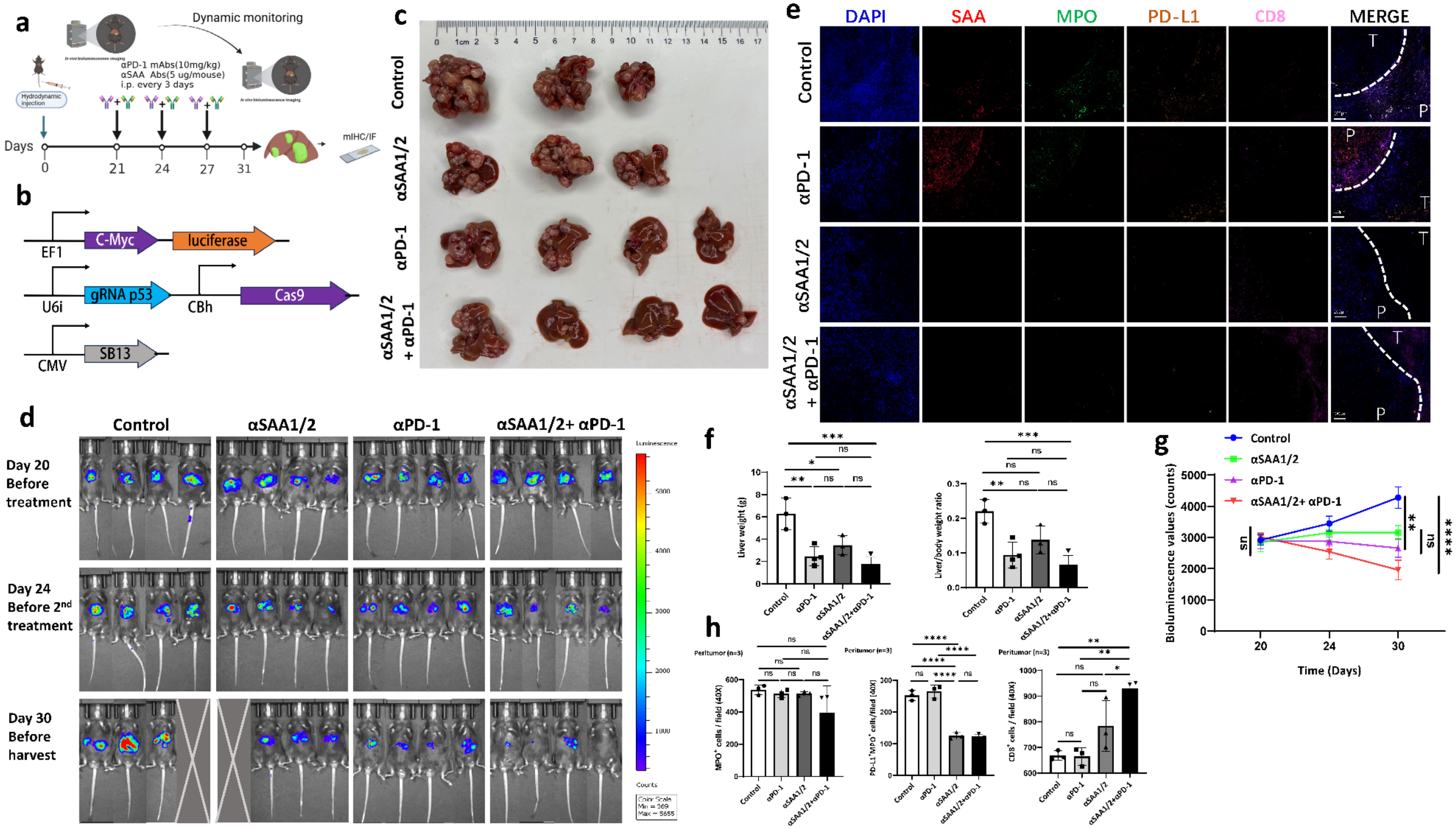

Supplementary Fig. 15. αSAA1/2 enhances αPD-1 efficacy in hydrodynamic injection HCC mice models. (a) Hydrodynamic injection murine models were separately treated with PBS combined with 0.5% methylcellulose, αPD-1 (10 mg/kg), αSAA1/2 (5 µg/mice), or αPD-1 (10 mg/kg) combined with αSAA1/2 (5 µg/mice) per three days. n = 4 mice. After 31 days, the mice were sacrificed and necropsied. The whole body and organs of mice were weighed after treatment. (b) The three gene constructs encoding injected in the hydrodynamic injection model. (c) Images of tumor tissue samples were taken from the necropsied orthotopic mice model (One of each died in the control and αSAA1/2 group when sacrificed). n = 3, 3, 4, 4 mice separately. (d) Before treatment, the 2nd treatment and harvest, luciferase marker expression was dynamically detected using IVIS. n = 4 mice. (e) The confocal microscopy images showed the expression of SAA, PD-L1, MPO^+ neutrophils and $CD8^+$ T cells in each treatment group by immunofluorescence analysis. (f) Histograms showed weight changes in the liver weight and liver to body weight ratio in different treatment groups. n = 3, 3, 4, 4 mice separately. (g) The bioluminescence value for each mouse over time with associated statistical analyses. n = 4 mice. (h) The counts of MPO^+ cells, $PD\text{-}L1^+MPO^+$ cells and $CD8^+$ T cells in peritumor region were analyzed by mIHC in each group. n = 3 mice. CD8: pink, DAPI: blue, MPO: green, PD-L1: orange, and SAA: red. Statistical data presented in this figure show mean ± SEM. ns indicates $P > 0.05$, $^{**}P < 0.01$, and $^{****}P < 0.0001$, by one-way ANOVA (f, g, h). Source data and exact *P* values are provided as a Source Data file. Illustrations created with BioRender.com.

Supplementary Fig. 16

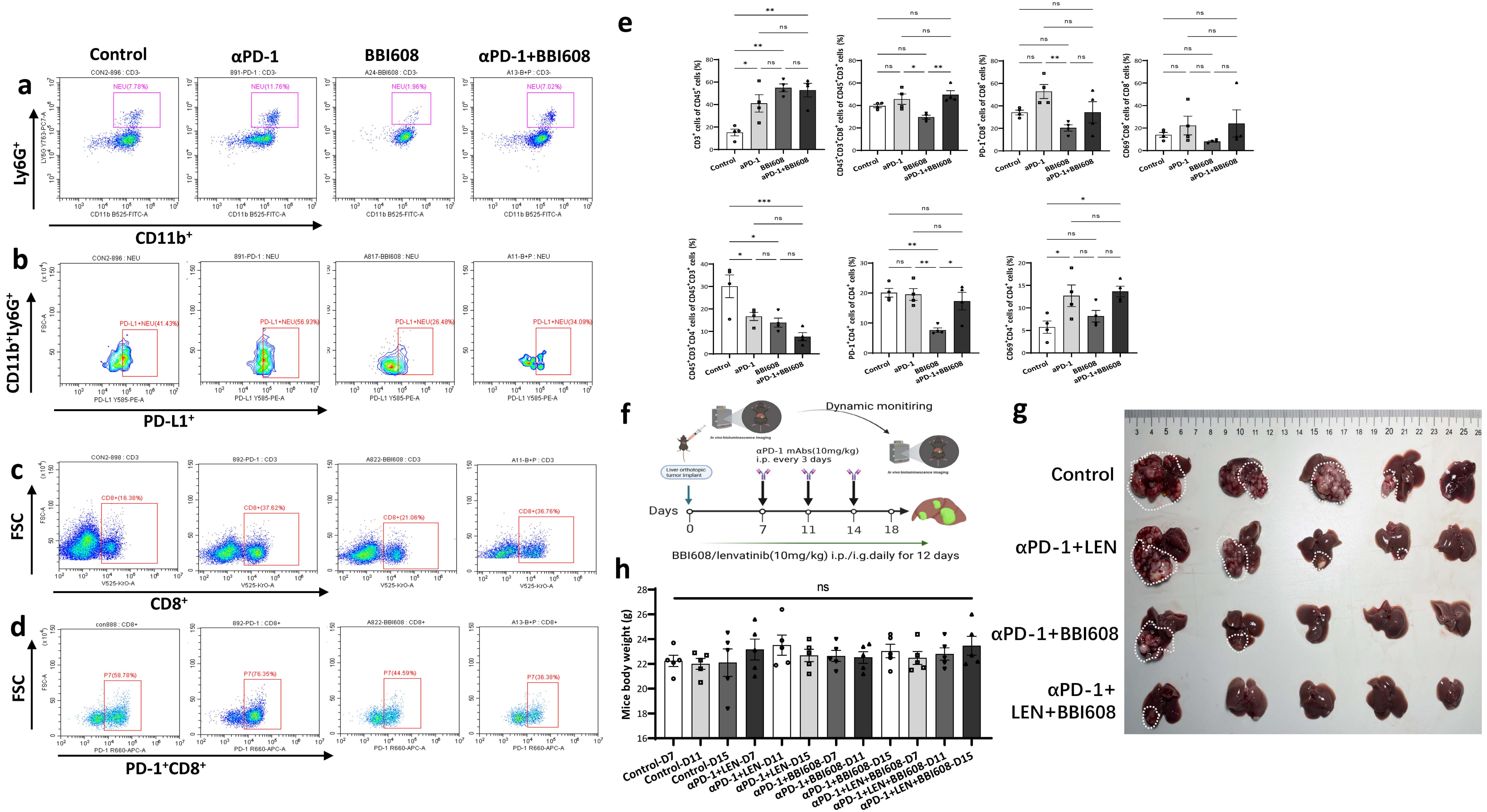

Supplementary Fig. 16. The immune cell infiltration in HCC mice models of αPD-1 combined with STAT3 inhibition and αPD-1 shows synthetic anti-HCC activity in combination with TKIs, STAT3 inhibitor, or both *in vivo*. (a-d) Flow cytometry images show the parent percentages of peritumoral infiltration of $CD11b^+Ly6G^+$ neutrophils, $PD\text{-}L1^+CD11b^+Ly6G^+$ neutrophils, $CD8^+$ T cells, and $PD\text{-}1^+CD8^+$ T cells in treatment groups including control, αPD-1, BBI608, and the combination of αPD-1 and BBI608. n = 4 mice. (e) Histograms represent the statistic comparison of the parent percentages of peritumoral infiltration of $CD45^+CD3^+$ cells, $CD45^+CD3^+CD8^+$ T cells, $CD45^+CD3^+CD8^+PD\text{-}1^+$ T cells, $CD45^+CD3^+CD8^+CD69^+$ T cells, $CD45^+CD3^+CD4^+$ T cells, $CD45^+CD3^+CD4^+PD\text{-}1^+$ T cells, and $CD45^+CD3^+CD4^+CD69^+$ T cells in each treatment group. n = 4 mice. (f) Orthotopic Hepa1-6-$luci^+$ mice were separately treated with PBS combined with DMSO, αPD-1 (10 mg/kg, per three days) combined with lenvatinib (10 mg/kg, daily), αPD-1 (10 mg/kg, per three days) combined with BBI608 (10 mg/kg, daily), or αPD-1 (10 mg/kg, per three days) combined with lenvatinib (10 mg/kg, daily) and BBI608 (10 mg/kg, daily). n = 5 mice. After 18 days, the mice were sacrificed and necropsied. The whole body of mice was weighed after treatment. (g) Images of tumor tissue samples were taken from the necropsied orthotopic mice model in each group. n = 5 mice. (h) Histograms showed weight changes in the whole mice in different treatment groups. n = 5 mice. Statistical data presented in this figure show mean ± SEM. ns indicates $P > 0.05$, and $^{**}P < 0.01$, by one-way ANOVA (e, h). Source data and exact *P* values are provided as a Source Data file. Illustrations created with BioRender.com.
