# Supplementary material for: Serum amyloid A promotes glycolysis of neutrophils during PD-1 blockade resistance in hepatocellular carcinoma
Source: Nat Commun. 2024 Feb 26;15:1754. doi: 10.1038/s41467-024-46118-w (PMC10897330; doi:10.1038/s41467-024-46118-w)
Supplement: Supplementary file 3 — Reporting Summary [file 41467_2024_46118_MOESM3_ESM.pdf]

nature portfolio

Corresponding author(s): Ning Lyu & Ming Zhao

Last updated by author(s): Jan 31, 2024

Reporting Summary

Nature Portfolio wishes to improve the reproducibility of the work that we publish. This form provides structure for consistency and transparency in reporting. For further information on Nature Portfolio policies, see our Editorial Policies and the Editorial Policy Checklist.

Statistics

For all statistical analyses, confirm that the following items are present in the figure legend, table legend, main text, or Methods section.

n/a	Confirmed	
☐	☒	The exact sample size (*n*) for each experimental group/condition, given as a discrete number and unit of measurement
☐	☒	A statement on whether measurements were taken from distinct samples or whether the same sample was measured repeatedly
☐	☒	The statistical test(s) used AND whether they are one- or two-sided<br>*Only common tests should be described solely by name; describe more complex techniques in the Methods section.*
☐	☒	A description of all covariates tested
☐	☒	A description of any assumptions or corrections, such as tests of normality and adjustment for multiple comparisons
☐	☒	A full description of the statistical parameters including central tendency (e.g. means) or other basic estimates (e.g. regression coefficient) AND variation (e.g. standard deviation) or associated estimates of uncertainty (e.g. confidence intervals)
☐	☒	For null hypothesis testing, the test statistic (e.g. *F*, *t*, *r*) with confidence intervals, effect sizes, degrees of freedom and *P* value noted<br>*Give P values as exact values whenever suitable.*
☒	☐	For Bayesian analysis, information on the choice of priors and Markov chain Monte Carlo settings
☒	☐	For hierarchical and complex designs, identification of the appropriate level for tests and full reporting of outcomes
☐	☒	Estimates of effect sizes (e.g. Cohen's *d*, Pearson's *r*), indicating how they were calculated

Our web collection on statistics for biologists contains articles on many of the points above.

Software and code

Policy information about availability of computer code

Data collection

For RNA-seq, human tumor tissues and neutrophils were collected and sent to Novogene(en.novogene.com) for RNA extraction and Illumina sequencing. The levels of the cytokines were analyzed by ScanArray Express (Perkinelmer, USA).Plasma concentration of SAA protein was measured using a commercially available Kit For serum amyloid A protein Assay (Purebio Biotechnology, China). Level of serum CRP and ALB were quantified using the NANOPIA CRP kit (SEKISUI MEDICAL, Japan) and the Albumin Gen.2 (ALB2) kit (Roche Diagnostics GmbH, Germany), respectively. Blood routine examinations were performed with the SYSMEX XN-2000 automatic blood analyzer.The rate of glucose uptake and lactate secretion was quantified using Multiparameter Bioanalytical System (YSI 2900, YSI Life Sciences) in 96-well format and analyzed as the previous study described(Deng, H. et al. J Immunother Cancer 9, doi:10.1136/jitc-2020-002305.).Glycolysis of neutrophils was analyzed through the Seahorse XFe96Analyzer (Agilent, California, USA). Images were captured using Vectra Polaris™ Automated Quantitative Pathology Imaging System (Akoya Biosciences, USA).The super-resolution images were obtained by Airyscan 2 with a confocal laser scanning microscope (LSM980, ZEISS; 63×/1.40 oil objective with 2.5x digital zoom, 35 nm/px, XY < 120 nm, Z < 350 nm).For The Cancer Genome Atlas (TCGA) cohort, clinical and RNA-seq data related to 424 HCC samples (including 50 paired tumor and peritumor normal tissues) were collected from the FireBrowse data portal.

Data analysis	Differential expression analysis was performed using the DESeq2 R package (1.16.1). Principal Component Analysis (PCA) was implemented by the ggplot2 R package. Gene ontology enrichment analysis was implemented by the clusterProfiler R package. Gene ontology terms with corrected P< 0.05 were considered significantly enriched by differentially expressed genes. We used the single-sample gene set enrichment analysis (ssGSEA) algorithm implemented in the R package's gene set variation analysis (GSVA) to calculate the enrichment scores of immune cell type infiltration. Positive stain quantification and spatial cell analysis were performed using HALO® image analysis software (Indica Labs, USA, version 2.3.2089.34).

For manuscripts utilizing custom algorithms or software that are central to the research but not yet described in published literature, software must be made available to editors and reviewers. We strongly encourage code deposition in a community repository (e.g. GitHub). See the Nature Portfolio guidelines for submitting code & software for further information.

Data

Policy information about availability of data

All manuscripts must include a data availability statement. This statement should provide the following information, where applicable:

- Accession codes, unique identifiers, or web links for publicly available datasets
- A description of any restrictions on data availability
- For clinical datasets or third party data, please ensure that the statement adheres to our policy

All study data are presented in the manuscript and supplementary materials. All the relevant raw data that support the findings of this study have been deposited in the Research Data Deposit public platform under the accession number RDDB2023340106 [https://www.researchdata.org.cn/Search.aspx?k=RDDB2023340106]. The raw sequence data reported in this paper have been deposited in the Genome Sequence Archive under the accession number HRA005809 [https://ngdc.cncb.ac.cn/search/?dbId=hra&q=HRA005809]. Source data are provided with this paper.

Research involving human participants, their data, or biological material

Policy information about studies with human participants or human data. See also policy information about sex, gender (identity/presentation), and sexual orientation and race, ethnicity and racism.

Reporting on sex and gender	We reported sex of all patients enrolled in the study in Supplementary Table 1-6. No gender related issues are applied to the analysis.
Reporting on race, ethnicity, or other socially relevant groupings	All the patients included are Chinese people who belong to the yellow race.
Population characteristics	All patients were definitely diagnosed as hepatocellular carcinoma. Detailed patients characteristics were provided in Supplementary Table 1-6.
Recruitment	All the HCC patients enrolled in the study were collected retrospectively, and we collected the medical data from the medical record system. There was no self-selection bias in this study.
Ethics oversight	The study was approved by the Ethical Review Committee of Sun Yat-sen University Cancer Center (Guangzhou, China; approval number: GZR2017-236 and B2023-688-01). All the patients who took part in the trial provided written informed consent.

Note that full information on the approval of the study protocol must also be provided in the manuscript.

Field-specific reporting

Please select the one below that is the best fit for your research. If you are not sure, read the appropriate sections before making your selection.

☒ Life sciences ☐ Behavioural & social sciences ☐ Ecological, evolutionary & environmental sciences

For a reference copy of the document with all sections, see nature.com/documents/nr-reporting-summary-flat.pdf

Life sciences study design

All studies must disclose on these points even when the disclosure is negative.

Sample size	No statistical methods were used to predetermine sample size. Yet sample size of animal experiments was estimated on the basis of similar research reported in the literature. In most of the experiments, 3 to 10 mice/samples were sufficient to identify differences between groups with at least 80% power and a 5% significance level. For clinical sample analysis, sample size was determined on the basis of similar research reported in the literature. For in vitro experiments, the sample size was determined based on pilot experiments or previous studies. All experiments included at least 3 independent experiments. The number of independent experiments was indicated in each figure legend.
Data exclusions	No data was excluded.
Replication	For each experiments of the number of biological independent animal/sample/patients is reported in the figure legend. For in vitro experiments at least three biological replicates were performed with similar results. For in vivo studies at least 3 animals were allocated per group.

Randomization	The samples used in this study were randomly allocated into control or experimental groups.
Blinding	For in vivo studies, the tumor measurement, treatment and analysis were performed blindly by different researchers. For in vitro studies, cells were treated identically without prior designation.

Reporting for specific materials, systems and methods

We require information from authors about some types of materials, experimental systems and methods used in many studies. Here, indicate whether each material, system or method listed is relevant to your study. If you are not sure if a list item applies to your research, read the appropriate section before selecting a response.

Materials & experimental systems

n/a	Involved in the study
☐	☒ Antibodies
☐	☒ Eukaryotic cell lines
☒	☐ Palaeontology and archaeology
☐	☒ Animals and other organisms
☐	☒ Clinical data
☒	☐ Dual use research of concern
☒	☐ Plants

Methods

n/a	Involved in the study
☒	☐ ChIP-seq
☐	☒ Flow cytometry
☒	☐ MRI-based neuroimaging

Antibodies

Antibodies used

Anti-mouse PD-1 (clone RMP1-14, rat, monoclonal) Bio X Cell Cat# BE0146 RRID: AB_10949053 Immunotherapy treatment (1 mg/mL) 10 mg/kg twice a week for 2 weeks
Anti-mouse SAA (clone 67604, Goat, Polyclonal) R&D Systems Cat# AF2948 RRID: AB_2182774 Anti SAA treatment (25 µg/mL) 5 µg/mouse twice a week for 2 weeks
Anti-Human/Mouse Myeloperoxidase (Goat, Polyclonal) R&D Systems Cat# AF3667 RRID: AB_2250866 IF (1:50)
Anti-Human IL-6 (clone 6708, mouse, monoclonal) R&D Systems Cat# MAB206 RRID: AB_2127617 mIHC (1:200)
Anti-mouse SAA1 + SAA2 (clone EPR19235, rabbit, monoclonal) Abcam Cat# ab199030 RRID: unregistered yet IHC (1:1000); IF (1:500)
Anti-human SAA1 + SAA2 (clone EPR19550, rabbit, monoclonal) Abcam Cat# ab207445 RRID: unregistered yet mIHC (1:1000); IF (1:500); WB (1:5000)
Anti-PD-L1 (clone SP142, rabbit, monoclonal) Abcam Cat# ab228462 RRID: AB_2827816 mIHC (1:200); IF (1:50)
Anti-PD-L1 (clone D5V3B, rabbit, monoclonal) Cell Signaling Technology Cat# 64988S RRID: AB_2799672 IHC (1:200); IF (1:50)
Anti-Stat3 (clone 124H6, mouse, monoclonal) Cell Signaling Technology Cat# 9139T RRID: unregistered yet WB (1:1000)
Anti-Phospho-Stat3 (Tyr705) (clone D3A7 Rabbit, monoclonal) Cell Signaling Technology Cat# 9145T RRID: unregistered yet WB (1:2000)
Anti-β-Actin (clone 13E5, Rabbit, monoclonal) Cell Signaling Technology Cat# 4970S RRID: AB_2223172 WB (1:1000)
Anti-PKM2 (clone D78A4, rabbit, monoclonal) Cell Signaling Technology Cat# 4053S RRID: AB_1904096 WB (1:1000)
Anti-LDHA (C4B5, rabbit, monoclonal) Cell Signaling Technology Cat# 3582S RRID: AB_2066887 WB (1:1000)
Anti-MCT4 (rabbit, polyclonal) Proteintech Cat# 22787-1-AP RRID: AB_11182479 WB (1:2000)
Anti-GAPDH (D16H11, rabbit, monoclonal) Cell Signaling Technology Cat# 5174S RRID: AB_10622025 WB (1:1000)
Anti-human CD15 (clone MMA + BY87rabbit, monoclonal) ZSGB-Bio Cat# ZM-0037 RRID: unregistered yet mIHC (1:200)
Anti-human CD68 (clone KP1, rabbit, monoclonal) ZSGB-Bio Cat# ZM-0060 RRID: AB_2904190 mIHC (1:200)
Anti-human CD8 (clone SP16, rabbit, monoclonal) ZSGB-Bio Cat# ZA-0508 RRID: AB_2890107 mIHC (1:200)
Anti-human OSM (clone G-1, mouse, monoclonal) Santa Cruz Biotechnology Cat# Sc-390253 WB (1:1000)
Anti-human Arginase 1 (rabbit, polyclonal) GeneTex Cat# GTX109242 RRID: AB_2036264 WB (1:500)
Anti-IL-1β (clone 3A6, mouse, monoclonal) Cell Signaling Technology Cat# 12242S RRID: AB_2715503 WB (1:1000)
Anti-mouse IFN-γ (clone H22, Armenian Hamster, monoclonal) BioLegend Cat# 513202 RRID: AB_1089144 IF (1:50)
Donkey anti-Goat IgG (H+L) Cross-Adsorbed Secondary Antibody, Alexa Fluor 488 Invitrogen Cat# A-11055 RRID: AB_2534102 IF (1:500)
Donkey anti-Rabbit IgG (H+L) Highly Cross-Adsorbed Secondary Antibody, Alexa Fluor 594 Invitrogen Cat# A-21207 RRID: AB_141637 IF (1:500)
Goat Anti-Mouse IgG H&L (Alexa Fluor 647) Abcam Cat# ab150115 RRID: AB_2687948 IF (1:500)
Ultra-LEAF Purified anti-human CD28 BioLegend Cat# 302934 RRID: AB_11148949 T cell stimulation (2 ug/mL)
Ultra-LEAF Purified anti-human CD3 BioLegend Cat# 317326 RRID: AB_11150592 T cell stimulation (2 ug/mL)
APC anti-human CD16 BioLegend Cat# 302012 RRID: AB_314212 Flow cytometry (100×)
FITC anti-human CD66b BioLegend Cat# 305104 RRID: AB_314496 Flow cytometry (100×)
Brilliant Violet 421 anti-human CD274 (B7-H1, PD-L1) BioLegend Cat# 329714 RRID: AB_2563852 Flow cytometry (100×)
PE anti-human TNF BioLegend Cat# 376203 RRID: AB_2894502 Flow cytometry (100×)
PE anti-human IFN-γ BioLegend Cat# 502508 RRID: AB_315233 Flow cytometry (100×)
PE anti-human CD274 (B7-H1, PD-L1) BioLegend Cat# 329706 RRID: AB_940368 Flow cytometry (100×)
Alexa Fluor 647 Mouse Anti-Human GLUT1 BD Biosciences Cat# 566580 RRID: AB_2869787 Flow cytometry (100×)
Purified anti-mouse CD16/32 Antibody BioLegend Cat# 101301 RRID: AB_312800 Flow cytometry (100×)

	Cell Activation Cocktail (with Brefeldin A) BioLegend Cat#423303 Flow cytometry (100×)<br>Alexa Fluor 700 anti-mouse CD45 BioLegend Cat# 103128 RRID: AB_493715 Flow cytometry (100×)<br>PerCP/Cyanine5.5 anti-mouse CD3 BioLegend Cat# 100218 RRID: AB_1595492 Flow cytometry (100×)<br>Brilliant Violet 605 anti-mouse CD4 BioLegend Cat# 100451 RRID: AB_2564591 Flow cytometry (100×)<br>Brilliant Violet 421 anti-mouse CD25 BioLegend Cat# 102033 RRID: AB_10895908 Flow cytometry (100×)<br>FITC anti-mouse CD3ε BioLegend Cat# 100306 RRID: AB_312671 Flow cytometry (100×)<br>Brilliant Violet 510 anti-mouse CD8a BioLegend Cat# 100752 RRID: AB_2563057 Flow cytometry (100×)<br>Alexa Fluor 700 anti-mouse/human CD11b BioLegend Cat# 101222 RRID: AB_493705 Flow cytometry (100×)<br>APC anti-mouse F4/80 BioLegend Cat# 123116 RRID: AB_893481 Flow cytometry (100×)<br>Brilliant Violet 650 anti-mouse Ly-6C BioLegend Cat# 128049 RRID: AB_2800630 Flow cytometry (100×)<br>PE/Cyanine7 anti-mouse Ly-6G BioLegend Cat# 127618 RRID: AB_1877261 Flow cytometry (100×)<br>Brilliant Violet 421 anti-mouse Ly-6G/Ly-6C (Gr-1) BioLegend Cat# 108445 RRID: AB_2562903 Flow cytometry (100×)<br>PE anti-mouse CD279 (PD-1) BioLegend Cat# 135206 RRID: AB_1877231 Flow cytometry (100×)<br>Brilliant Violet 785 anti-mouse TNF BioLegend Cat# 506341 RRID: AB_2565951 Flow cytometry (100×)<br>Alexa Fluor 647 anti-mouse/rat/human FOXP3 BioLegend Cat# 320013 RRID: AB_439749 Flow cytometry (100×)<br>PE/Cyanine7 anti-mouse CD279 (PD-1) BioLegend Cat# 135215 RRID: AB_10696422 Flow cytometry (100×)<br>Brilliant Violet 421 anti-mouse CD69 BioLegend Cat# 104545 RRID: AB_2686969 Flow cytometry (100×)<br>APC anti-human CD15 (SSEA-1) BioLegend Cat# 301908 RRID: AB_314200 Flow cytometry (100×)<br>Zombie NIR Fixable Viability Kit BioLegend Cat# 423105 Flow cytometry<br>Zombie Red Fixable Viability Kit BioLegend Cat# 423109 Flow cytometry
Validation	All antibodies were purchased from reputable commercial vendors with provided validation information.

Eukaryotic cell lines

Policy information about cell lines and Sex and Gender in Research

Cell line source(s)	Liver cell lines HHL-5 (kindly provided by Prof. Bo Li, Sun Yat-sen University) derived from human hepatocytes were used for in vitro experiments. The murine HCC cell lines Hepa1-6-luc+ were purchased form Procell used for in vivo experiments. Neutrophils or T cells were isolated from the blood of patients with advanced HCC (aHCC) treated at the Sun Yat-sen Cancer Center or healthy donors.
Authentication	All the cell lines were authenticated by short tandem repeat profiling prior to use.
Mycoplasma contamination	All the cell lines were tested for mycoplasma contamination by PCR every two months, only mycoplasma negative cells were used for experiments.
Commonly misidentified lines<br>(See ICLAC register)	No commonly misidentified cell lines were used.

Animals and other research organisms

Policy information about studies involving animals; ARRIVE guidelines recommended for reporting animal research, and Sex and Gender in Research

Laboratory animals	WT male and female C57BL/6J mice were purchased from the Guangdong Medical Laboratory Animal Center, and Saa1 knockout mice were purchased from Cyagen Biosciences Inc (Guangzhou, China). Stat3flox/flox mice were purchased from Jackson Laboratory and bred to Alb-cre+/+ mice to generate Stat3flox/flox & Alb-cre+/- mice.<br>All mice were housed and treated in the animal facility of Sun Yat-sen University Cancer Center or Sun Yat-sen University. All mice were maintained under specific pathogen-free (SPF) conditions and were used between 6 and 8 weeks of age. All mice were housed five per cage under a 12-h light-dark cycle (light on from 7 a.m. to 7 p.m., humidity between 30% and 70%, temperatures of 20–22 °C) with free access to food and water. Mice were monitored three times per week for general health and euthanized early based on defined endpoint criteria, including ascites, lethargy, or other signs of sickness or distress. If animals appeared moribund or the diameter of the tumors reached 15 mm, the mice were sacrificed. In some cases, the maximal tumor burden permitted has been exceeded the last day of measurement and the mice were immediately euthanized. Mice were age- and gender-matched with appropriate control mice for analysis.
Wild animals	This study did not involve wild animals.
Reporting on sex	The findings apply to both sexes. Both male and female mice were used in this study. No selection for sex of mice was performed.
Field-collected samples	This study did not involve field-collected samples.
Ethics oversight	All animal procedures have been done according to the institutional guidelines and approved by the local ethics committee (approval number: L102012017003I).

Note that full information on the approval of the study protocol must also be provided in the manuscript.

Clinical data

Policy information about clinical studies

All manuscripts should comply with the ICMJEguidelines for publication of clinical research and a completedCONSORT checklist must be included with all submissions.

Clinical trial registration	A retrospective study
Study protocol	The study protocol in this paper are available from the corresponding authors upon request.
Data collection	We retrospectively screened 569 patients who received anti-PD-1-based immunotherapies either as single or combined with tyrosine kinase inhibitors (TKIs), locoregional procedures, or both between April 2017 and December 2019. A total of 243 patients were excluded because of missing image examination (n = 109), uncompleted follow-up (n = 79), without target tumors for radiological assessment (n = 24), missing written informed consents of patients (n = 17), secondary malignancies (n = 9), and treated with traditional Chinese medicine along with anti-PD-1 agent (n = 5). Among the remaining 326 patients, 52 patients were treated with single PD-1 inhibitors, 99 patients were treated with PD-1 inhibitors plus TKIs, 97 patients were treated with PD-1 inhibitors plus locoregional procedures (including transarterial chemoembolization and transarterial infusion of chemotherapy), and 78 patients were treated with a combination of PD-1 inhibitors, TKIs, and locoregional procedures (Supplementary Fig. 1a). Furthermore, in order to validate our findings, we conducted a retrospective collection of data from 138 patients who received anti-PD-1-based immunotherapies combined with tyrosine kinase inhibitors (TKIs), as well as locoregional procedures, or both, between January 2021 to January 2023 (Supplementary Table 5). Patient's epidemiologic characteristics, treatment strategies, and follow-up information were obtained from a prospective clinical database of SYSUCC. All therapies were performed according to the recommendations of clinical trials with high-level of evidence. Response assessment by computed tomographic or magnetic resonance imaging was performed every 6–8 weeks in the first year of treatment and every 8–12 weeks thereafter. After two years of anti-PD-1 treatment, patients were recommended to stop immunotherapy and contacted every 8–12 weeks to document the survival status. Survival outcomes were defined as follows: overall survival (OS) was defined as the time between randomization date and death of any cause and progression-free survival PFS was defined as the time between randomization date and disease progression or death of any cause. Radiological tumor response assessment was conducted according to the Response Evaluation Criteria in Solid Tumors (RECIST) version 1.1. The follow-up data of the derivation cohort was censored at the time of July 15, 2021, while the validation cohort was censored at the time of July 20, 2023.
Outcomes	Results: SAA correlates with the efficacy of PD-1 blockade in aHCC patients

Plants

Seed stocks	*Report on the source of all seed stocks or other plant material used. If applicable, state the seed stock centre and catalogue number. If plant specimens were collected from the field, describe the collection location, date and sampling procedures.*
Novel plant genotypes	*Describe the methods by which all novel plant genotypes were produced. This includes those generated by transgenic approaches, gene editing, chemical/radiation-based mutagenesis and hybridization. For transgenic lines, describe the transformation method, the number of independent lines analyzed and the generation upon which experiments were performed. For gene-edited lines, describe the editor used, the endogenous sequence targeted for editing, the targeting guide RNA sequence (if applicable) and how the editor was applied.*
Authentication	*Describe any authentication procedures for each seed stock used or novel genotype generated. Describe any experiments used to assess the effect of a mutation and, where applicable, how potential secondary effects (e.g. second site T-DNA insertions, mosiacism, off-target gene editing) were examined.*

Flow Cytometry

Plots

Confirm that:

- [x] The axis labels state the marker and fluorochrome used (e.g. CD4-FITC).
- [x] The axis scales are clearly visible. Include numbers along axes only for bottom left plot of group (a 'group' is an analysis of identical markers).
- [x] All plots are contour plots with outliers or pseudocolor plots.
- [x] A numerical value for number of cells or percentage (with statistics) is provided.

Methodology

Sample preparation	Flow cytometry experiments were performed on cell lines and tumor samples. For in vitro experiments, cells were collected, washed in PBS and stained with antibodies according to standard procedures. For in vivo experiments, to create cell suspensions, tumors were removed, finely chopped, and suspended in PBS. Tumors were digested with collagenase/Dispase for 30min at 372C then filtered through a 40 um mesh. Cells were resuspended in red blood cell lysis buffer for 1min at room temperature. Cells were resuspended in PBS, counted, Fc receptors were blocked with CD16/32 Ab (Biolegend),and then 1 million cells were stained with coniugated Ab cocktail for 20min on ice Cells were washed two times in PBSand then resuspended for flow cytometry analysis.

Instrument	Flow cytometry analysis was performed on either a BD LSRFortessa, a BD FACSAria II (BD Biosciences).
Software	Data were collected using either BD FACSDiva software (BD Biosciences).Data were analyzed using FlowJo v10.
Cell population abundance	Purity of FACS-sorted samples was analyzed by flow cytometry. Purity of the samples was >90%.
Gating strategy	Starting cells were gated by FSC/SSC gates. Gates indicating boundaries between "positive" and "negative" are according to the isotype staining. Expression of indicated proteins were checked on these populations as indicated in the figures and figure legends

☒ Tick this box to confirm that a figure exemplifying the gating strategy is provided in the Supplementary Information.
